# Biorefining of Anaerobic Digestates for the Recovery of Biostimulants and Bioelicitors for Immune Priming and Plant Protection

**DOI:** 10.1021/acs.est.5c03321

**Published:** 2025-09-30

**Authors:** Marco Greco, Daniele Coculo, Angela Conti, Savino Agresti, Daniela Pontiggia, Hugo Mélida, Lorenzo Favaro, Vincenzo Lionetti

**Affiliations:** † Department of Biology and Biotechnologies “Charles Darwin”, 9311 Sapienza University of Rome, Rome 00185, Italy; ‡ Department of Pharmaceutical Sciences, 9309 University of Perugia, Perugia 06123, Italy; § Agrolio s.r.l., S.P. 231 KM 55 + 120, Andria 70031, Italy; ∥ Research Center for Applied Sciences to the Safeguard of Environment and Cultural Heritage (CIABC), Sapienza University of Rome, Rome 00185, Italy; ⊥ Área de Fisiología Vegetal, Departamento de Ingeniería y Ciencias Agrarias, 16762 Universidad de León, León 24071, Spain; # Instituto de Biología Molecular, Genómica y Proteómica (INBIOMIC), Universidad de León, León 24071, Spain; ¶ Department of Agronomy Food Natural Resources Animals and Environment (DAFNAE), University of Padova, Agripolis, Legnaro, Padua 35020, Italy; ∇ Department of Microbiology, Stellenbosch University, Private Bag X1, Matieland 7602, South Africa

**Keywords:** olive pomace valorization, digestate metagenomics, plant immunity stimulation, circular waste upcycling, sustainable agriculture

## Abstract

Olive oil production is a major global agricultural industry that generates significant waste, particularly olive pomace, which poses environmental and economic challenges. Anaerobic digestion emerges as a promising solution for its valorization into biogas. However, the resulting digestate remains underutilized and its long-term environmental impact is uncertain. Traditional disposal methods are costly and inefficient, underscoring the need for more sustainable approaches. In this study, olive pomace digestate was biorefined, and its components were upcycled into soil amendments and plant immunostimulants. Metagenomic analysis revealed a diverse microbial community in the liquid fraction. A microbial-enriched protein extract (MIPE) was obtained, containing precursors of microbe- and damage-associated molecular patterns, including Flagellin, Elongation Factor Tu, and the plant phytocytokine Golven. Plant treatment with MIPE triggered a rapid immune response, characterized by oxidative burst, mitogen-activated protein kinase activation, and the upregulation of defense-related genes such as *CYP81F2*, *FRK1*, and *WRKY53*. MIPE-induced priming enhanced *Arabidopsis* and tomato resistance to *Botrytis cinerea* and *Pseudomonas syringae*. Our findings highlight olive pomace digestate as a valuable growth biostimulant, with its liquid fraction also representing a promising resource of immunity bioelicitors. This refinement valorizes olive mill waste, providing a sustainable alternative to chemical fertilizers and pesticides and supporting sustainable agriculture.

## Graphical abstract

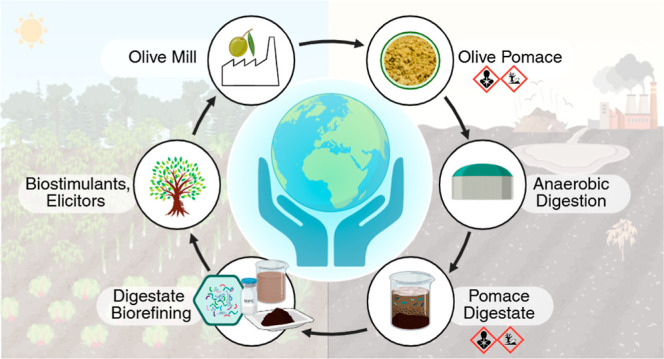

## 1 Introduction

A significant global increase in olive oil extraction and consumption has been recorded, driven by its appealing qualities and the growing recognition of its health benefits.^1^ Compared to traditional olive oil extraction methods, such as pressing and three-phase centrifugation, the two-phase system produces only two products, oil and wet pomace, without generating a separate wastewater phase.^2^ This process is more efficient and sustainable, yielding more oil, using less water, minimizing waste, and preserving the oil quality and nutritional properties. Two-phase olive pomace, the main byproduct of this system, is an acidic, organic-rich material consisting primarily of olive skin, pulp, stone fragments, water, and water-soluble compounds including oligosaccharides, polyphenols, and mineral elements.^3,4^ Its management remains challenging, as conventional methods such as composting and specialized treatments are costly.^5^ Consequently, olive pomace is often improperly disposed of on soil, which can exacerbate soil degradation and contribute to water eutrophication.^6,7^ Developing sustainable methods to convert olive pomace into valuable bioproducts is therefore crucial to reduce costs and minimize environmental impact.^3,4^ Anaerobic digestion (AD) is gaining attention as a promising biological solution for valorizing olive pomace by converting it into biogas and bioenergy.^8,9^ This process enhances the profitability of the olive oil industry while mitigating greenhouse gas emissions, as the CO_2_ released is biogenic and displaces petroleum-based fuels.^10–12^ As a result, more olive oil mills are adopting biogas plants to leverage olive pomace as a valuable biomass resource in anaerobic digestion systems.^13,14^ AD converts organic matter into biogas through hydrolysis, acidogenesis, acetogenesis, and methanogenesis, driven by bacteria and archaea.^15,16^ Alongside biogas, the process generates a semisolid or liquid residue known as digestate. While research has optimized AD process, digestate valorization remains underexplored.^17^

Digestates pose challenges due to its high moisture content, which increases volume and weight, complicates handling, transport, and nutrient utilization, and raises transportation costs.^18^ Incineration releases harmful carbon gases, contributing to pollution. Digestates could hinder the biogas industry’s growth without sustainable solutions and harm the environment.^19,20^ Digestates were proposed as a fertilizer because it contained partially degraded plant-derived organic matter, water, and essential nutrients.^21,22^ However, the nutrient composition of the digestate may be unbalanced. Its soil application can cause environmental issues, including ammonia and greenhouse gas emissions, nutrient leaching, pathogen spread, and micropollutant contamination.^19,23^ However, the digestate can harbor a diverse community of agronomically beneficial microorganisms, including plant growth-promoting bacteria, denitrifying and nitrifying bacteria, and nitrogen-fixing bacteria.^24^ Arbuscular mycorrhizal fungi and saprophytic fungi can also be present in digestates due to their resilience as spores or contamination from raw materials and the environment.^25^ However, the digestate may also contain pathogenic bacteria, opportunistic fungi, and antibiotic-resistant microbes, which could pose risks to plant health, soil quality, and even human and animal safety if not properly managed.^26^ The effects of olive pomace digestate on plant growth and productivity have not yet been investigated.

Interestingly, microbes and plant organic matter present in digestates may serve as elicitors to enhance plant immunity. This digestate valorization could be relevant for reducing reliance on chemical pesticides and minimizing health risks to workers and consumers.^27^ The plant immune system detects pathogenic microbes through pattern-recognition receptors (PRRs), which are either receptor kinases (RKs) or receptor proteins (RPs) located on the cell surface.^28^ These PRRs recognize conserved molecules from microbes, known as microbe-associated molecular patterns (MAMPs), triggering pattern-triggered immunity (PTI).^29,30^ This recognition initiates a signaling cascade that can result in the production of antimicrobial compounds, the reinforcement of the plant cell wall (CW), and activation of additional defense mechanisms to limit pathogen spread.^31–33^ MAMPs include fragments of flagellin (e.g., flg22), translation elongation factor EF-Tu, β-glucans, chitin, ergosterol, lipopolysaccharide, elicitin, and harpin.^34–39^ Importantly, danger signals can also arise from immunogenic plant host factors.^40^ The PRRs also detect endogenous danger molecules, including phytocytokines, cytosolic proteins, peptides, nucleotides, amino acids, and damage-associated molecular patterns (DAMPs), such as the oligosaccharides, from the degradation of the plant CW.^41^ Pectin fragments, such as oligogalacturonides (OG), are known elicitors.^42^ MAMPs’ and DAMPs’ application to plants can confer a greater ability to detect pathogens and activate defense responses faster than untreated crops.^43^ This can be a consequence of priming, a sensory state that enables plants to “remember” previous stress exposures and mount faster, stronger, and less energy-demanding defense responses improving plant resistance.^44,45^ This “primed state”, based on partial preactivation of immune pathways, can be induced by microbes, bioactive compounds, or chemicals, offering a sustainable way to enhance crop resilience.^43^ Nevertheless, excessive applications of such elicitors may induce a hyperimmune response, causing the plant to strike a costly growth-defense trade-off.^4,46^

Collectively, these considerations highlight both the environmental impact of olive pomace and its digestate and the largely unexplored potential of digestates as reservoirs of nutrients and bioactive compounds. Digestate upcycling could offer a novel route to integrate this underutilized byproduct into a circular economy pathway, converting it into valuable agricultural inputs while reducing dependence on synthetic fertilizers and pesticides. Therefore, this study aimed to develop a biorefining pipeline to recover and characterize bioactive fractions from olive pomace digestate, assess their nutrient composition, and evaluate their capacity to enhance plant growth and immunity. Specifically, this study proposes a biorefining approach to separate digestates into liquid and solid fractions, exploring their use as soil amendments, fertilizers, and immunostimulants. We characterized the biomass and nutrients and compared their ability to stimulate growth in *Arabidopsis* and tomato plants. The microbial communities were characterized by DNA metabarcoding. The liquid digestate was examined as a reservoir of low-cost MAMPs/DAMPs-based elicitors. A microbial-enriched protein extract (MIPE) was obtained from the liquid digestate and analyzed by proteomics to identify key immunogenic factors. MIPE was subsequently assessed for its ability to induce defense responses and protect *Arabidopsis* and tomatoes against pathogens.

## 2 Materials and Methods

### 2.1 Olive Pomace Digestate Collection and Fractionation

The raw digestate (RD) was collected from the AGROLIO-AGROENERGY biogas plant (Andria, BT, Italy), the first AD plant in the world to use olive pomace as sole feedstock. RD was generated under mesophilic conditions (45 °C) for 60 days using pomace derived from a two-phase olive oil extraction system processing olive (*Olea europaea* L., cultivar Coratina). RD (1L) was subjected to sedimentation overnight at 4 °C in a glass beaker to separate it into liquid digestate (LD) and solid digestate (SD). For testing the effects of digestate-derived microbes on plant growth and for metabarcoding analysis, LD was filtered through 25 μm Miracloth to remove residual plant particles and the filtrate was centrifuged at 5000*g* for 30 min at 4 °C, yielding a supernatant called microbial-depleted LD (MD-LD) and a microbial-enriched pellet (M). The M pellet was washed with sterile 1× PBS and used for the following experiments. The presence of microbial cells in M was assessed by light and fluorescence microscopy using fluorescein isothiocyanate and trypan blue staining (Nikon Eclipse E200, 40×).

### 2.2 MIPE Extraction and Protein Quantification

The protein/peptide fraction MIPE was obtained through sedimentation, centrifugation, and sonication steps, as outlined in a Patent publication.^47^ Briefly, the microbial pellet was resuspended in sterile water, and proteins were extracted via sonication (Sonics Vibra Cell VCX 130, Sonics & Materials, Inc., Newtown, CT, United States) at 70% amplitude, 5 min, 20 s ON/OFF pulses in ice bath. The lysate was centrifuged (15,000*g*, 30 min, 4 °C) to remove microbial and plant residues, and proteins were precipitated from supernatant with 20% trichloroacetic acid (Sigma Chemical Co, St. Louis, MO),^48^ centrifuged (6000*g*, 10 min, 4 °C), washed with hydrochloric acid-ethanol, lyophilized, and stored at −20 °C. MIPE yield was expressed as milligrams of dry weight per liter of LD. Lyophilized MIPE was dissolved in sterile distilled water at 5 mg/mL and used for protein analysis and biological assays. Protein concentration was determined by the Bradford method.^49^ The presence of residual nucleic acids in the extract was ruled out by spectrophotometry and agarose gel electrophoresis (1.5%, 60 V, 25 min). Images were captured using a Gel Doc XR + System (BioRad, Hercules, CA, USA) (Figure S5).

### 2.3 Plant Growth Conditions and Dose–Effect Treatments

*Arabidopsis thaliana* (ecotype Columbia, Col-0) seeds were surface-sterilized with 20% NaClO (5 min) and washed four times. After 2 days of stratification at 4 °C, seeds were germinated in multiwell plates (10 seeds/well) with 1 mL of liquid MS/2 medium (2.2 g/L MS, 0.5% sucrose, pH 5.7).^50^ To evaluate the dose effect of MIPE on *Arabidopsis* seedling growth, 7 day-old seedlings were treated with sterile water or MIPE (1, 10, or 100 μg dry mass/mL of distilled water) in MS/2 medium, where the extracts were sterilized using a MCE membrane filter (0.22 μm pore size). For measurements of shoot growth in adult plants, *Arabidopsis* seeds were initially germinated on solid MS/2 medium (2.2 g/L MS, 1% sucrose, 0.8% plant agar, pH 5.7) at 22 °C under a 16 h light/8 h dark cycle. After 7 days, seedlings were transferred to sterile soil and grown at 22 °C with a 12 h light/12 h dark cycle (photosynthetic active radiation 100 μmol m^–2^ s^–1^). A commercial soil was sterilized by autoclaving and amended with 45, 90, or 180 g/kg of RD (Figure 1A). Additional experiments tested 90 g/kg of RD vs 30 g/kg of SD or 60 g/kg of LD. Further treatments compared 30 g/kg of LD, 30 g/kg of MD-LD, and 1.5 g/kg of M. In all the experiments, rosette fresh and dry weights were measured after 2 weeks. For dry weight determination, the aerial vegetative portion of plants were weighed after treatment at 80 °C for 6 h.

**Figure 1:**
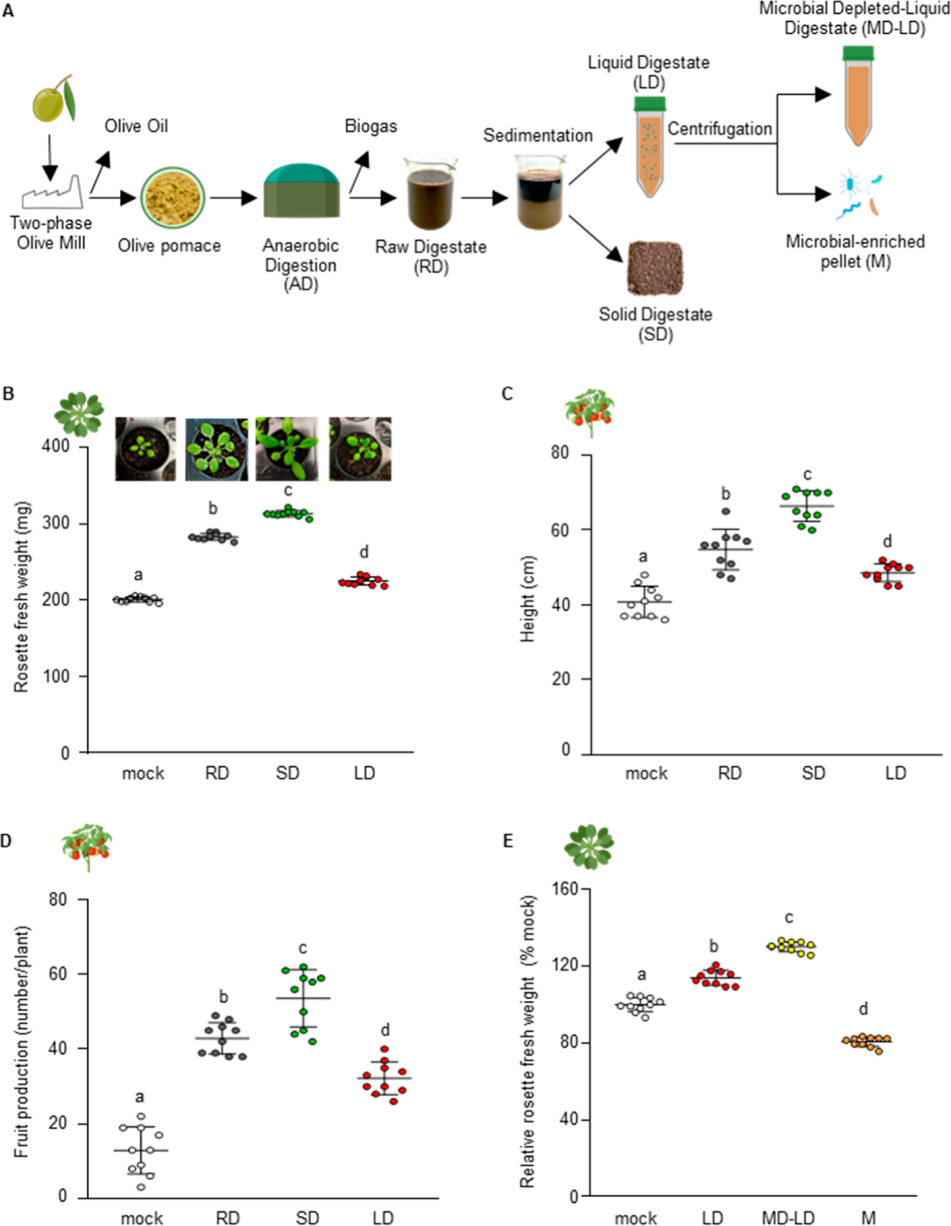
Biorefining of olive pomace digestate improves its biostimulant performance on *Arabidopsis* and tomato. (A) Diagram showing the process of preparation of the different fractions from the olive pomace raw digestate (RD). After sedimentation, the RD was fractionated into in solid digestate (SD) and liquid digestate (LD). After centrifugation, LD was fractionated in a microbe-depleted liquid digestate (MD-LD) and a microbial-enriched pellet (M). (B) Effects of soil amended with RD (45 g/kg), SD (15 g/kg), or LD (30 g/kg) fractions on *Arabidopsis* shoot growth, measured as rosette fresh weight. (C,D) Effects of soil amended with RD (20 kg/m^2^), SD (6 kg/m^2^), or LD (14 kg/m^2^) on height and fruit production of *S. lycopersicum*, grown on the field. (E) Effects of soil amended with LD, MD-LD (both 30 g/kg), or M (1.5 g/kg) on *Arabidopsis* shoot growth, measured as relative rosette fresh weight compared to mock. The values are expressed as percentages relative to plant grown on water-soaked soil used as mock. Icons next to the graphs indicate the plant species used for the analysis. Data shown represent the mean ± SD (*n* = 10). The experiments were repeated three times with similar results. The different letters indicate significantly different data sets according to ANOVA followed by Tukey’s test (*p* ≤ 0.05).

Tomato seeds (*Solanum lycopersicum*, cultivar Minibel) were purchased from Mascarell Semillas S.L., (Benissoda, Spain). Minibel is a determinate-growing cultivar producing small-sized fruits and well suited for laboratory-scale cultivation.^51^ Seeds were germinated on wet paper, transferred to soil, and grown in a greenhouse at 23 °C with a 16 h light/8 h dark cycle (photosynthetic active radiation 75 μmol m^–2^ s^–1^, 35–40% humidity). Five-week-old tomato plants were transplanted in soil with 20, 40, or 80 kg/m^2^ of RD. Tomato plants were also grown on the field with 20 kg/m^2^ of RD, 6 kg/m^2^ of SD, or 14 kg/m^2^ of LD. Height and fruit number were measured four months after transplantation. For both *Arabidopsis* and tomato experiments water-soaked soil was used as mock. Data shown represent the mean ± SD (*n* = 10). The experiments were repeated three times with similar results.

### 2.4 DNA Extraction and 16S and ITS rRNA Sequencing

The genomic DNA of microbial communities in M was extracted by using the DNeasy PowerSoil kit (QIAGEN, Germany). M was resuspended in UREA buffer, incubated at 60 °C, centrifuged, and processed according to the kit instructions. DNA concentration was assessed with NanoDrop1000 (ThermoFisher Scientific, MA, USA). Metagenomic DNA served as a template for 16S rRNA amplification using primers 8F/1492R.^52^ Polymerase chain reaction (PCR) was performed with Platinum SuperFi II PCR Master Mix (Invitrogen) under specific cycling conditions, and amplicons were validated on a 1% agarose gel.^53^ A tagging step with modified primers followed the Oxford Nanopore protocol (SQK-LSK114), with additional amplification, purification with Ampure XP, and barcoding. Sequencing libraries were prepared using a NEBNext Ultra DNA library preparation kit and loaded onto an R10.4.1 flow cell. Reads were processed with Guppy (v6.4.6), filtered (400–1800 bp), and aligned to the SILVA 16S database using minimap2. Taxonomic abundances were normalized using the geometric mean of pairwise ratios (GMPR) implemented in the *microeco* R package. Abundance data from 10 biological replicates were averaged (*n* = 10) and visualized with *ggplot2*. Community composition was assessed at the Genus, Family, Order, and Phylum levels. Sequencing data are available under BioProject ID PRJNA1211516 (https://www.ncbi.nlm.nih.gov/bioproject/?term=PRJNA1211516).

### 2.5 Protein Identification by LC–MS/MS Analysis

MIPE (15 μg of proteins) were prepared in 20 μL with 4× Laemmli buffer, denatured at 100 °C, and loaded onto a 12% acrylamide gel for 1D-SDS-PAGE. Proteins were visualized with Coomassie Brilliant Blue, each lane excised into seven gel slices, and subjected to in-gel trypsin digestion.^54^ Peptides were analyzed using ultrahigh-performance liquid chromatography and high-resolution mass spectrometry.^55^ Peptides were separated using a 75 μm C18 column (ES800-PepMap RSLC C18, 150 mm × 75 μm) and nano-LC (UltiMate 3000 RSLC, Thermo Fisher Scientific) with a 100 min gradient (4% to 90% eluent B, 80% acetonitrile, 0.1% formic acid, 0.3 μL/min flow). The analysis used a Q Exactive Plus Hybrid Quadrupole-Orbitrap mass spectrometer (Thermo Fisher Scientific) with data-dependent acquisition, selecting the 15 most intense ions. Full-scan spectra (*m*/*z* 350.0 to 1700.0, 70,000 ppm resolution) and higher-energy collisional dissociation (HCD) fragmentation (17,500 ppm) were used. Protein identification was performed with MaxQuant software v. 2.2.0.0 as previously described.^56,57^ A custom database (157,496 protein counts) was created for protein identification, combining UniProtKB reference proteomes for bacteria, fungi, and olives (released 09/05/2024), as detailed in Supporting Information Datasheet S1. The mass spectrometry data are available on the ProteomeXchange Consortium via the jPOST repository [http://repository.jpostdb.org], with data set identifier JPST003545/PXD059822.^58^ The proteomic analysis was performed on three biological replicates.

### 2.6 Digestate Characterization

Dry residue (total solids) was determined gravimetrically by drying fresh samples at 105 °C for 12 h to constant weight and expressed as percentage of the initial wet mass. Organic dry matter was measured by ignition of dried samples in a muffle furnace at 550 °C until complete incineration; ash content was calculated gravimetrically, and organic dry matter was obtained by difference (100–ash %). The pH of the LD was determined by using a CRISON GLP21 pH-meter (Hach Lange Spain, S.L.U., Barcelona, Spain). The content of nitrogen, ammonium, and ammonium nitrogen was evaluated according to the CNR IRSA methods.^59^ The contents of potassium, phosphorus, sulfur, magnesium, iron, copper, boron, manganese, nickel, chromium hexavalent, lead, cadmium, mercury, and zinc were measured by inductively coupled plasma–optical emission spectroscopy (ICP-OES) according to the UNI EN 16174:2012 + UNI EN 16170:2016 ^60,61^ methods. The detection of *Salmonella* spp. was performed according to APAT 3/Man 20.^62^ The monosaccharide composition and the oligosaccharide characterization are described in Text S1.

### 2.7 Hydrogen Peroxide Quantification

Four millimeter diameter leaf discs from four-week-old *Arabidopsis* plants and five-week-old *S. lycopersicum* were used to determine hydrogen peroxide (H_2_O_2_) production. Specifically, leaf discs were placed per well in a white 96-well plate and were incubated overnight with 150 μL of distilled water at room temperature. The following day, distilled water was replaced by 100 μL of 10 nM L-012, a chemical analogue of luminol (FUJIFILM Wako Pure Chemical Corporation), and 10 μg/mL horseradish peroxidase (HRP; Sigma-Aldrich), and incubated for 2 h. Subsequently, leaf discs were treated with 1 μg/mL MIPE or 1 μM flg22 (synthetic 22–amino acid flagellin-derived peptide, PhytoTech laboratories). Sterile distilled water was used as a mock. H_2_O_2_ production was measured for 200 min by determining the luminescence produced by the luminol-peroxidase reaction in a Varioskan Lux luminescence reader (Thermo Scientific, Waltham, MA, USA).^63^ For each experiment, six leaf discs were used, each collected from a different adult plant (*n* = 6). The experiment was performed three times with similar results.

### 2.8 Immunoblot Analysis for Mitogen-Activated Protein Kinases Activation

Ten-day-old *Arabidopsis* seedlings were treated with MIPE (2 μg/mL), flg22 (1 μM), or sterile distilled water was used as a mock for 5, 10, and 20 min and then immediately frozen in liquid nitrogen. Protein extraction and detection of activated mitogen-activated protein kinases (MAPKs) were performed as previously described.^63^ Briefly, frozen seedlings were homogenized, and proteins were extracted using a Tris-HCl-based buffer with protease and phosphatase inhibitors. Total proteins were quantified by the Bradford assay, separated by SDS-PAGE, and transferred onto nitrocellulose membranes. Membranes were blocked, incubated with anti-Phospho-p44/42 MAPK primary antibody (Cell Signaling Technology Danvers, MA, USA) overnight at 4 °C, washed, incubated with HRP-conjugated secondary antibody (Thermo Fisher Scientific, Waltham, MS, USA), and developed using ECL. Equal loading was confirmed by Ponceau S staining. The experiment was performed three times with similar results.

### 2.9 *Arabidopsis* and Tomato Infection Assay

*Botrytis cinerea* (strain SF1 ^64^) was grown in the dark before conidial collection at 23 °C and 70% relative humidity for 20 days on malt extract agar (20 g/L) with mycological peptone (10 g/L) and micro agar (12 g/L). Conidia of *B. cinerea* were collected by washing the mycelium from agar plates with sterile water, filtered, and counted using a Thoma counting chamber. Four-week-old plants were pretreated with 2 mL of flg22 (1 μM) or MIPE (2 μg/mL) and sterile distilled water. After 24 h, each leaf was infected with six 5 μL droplets containing 1 × 10^6^ conidia/mL in Potato Dextrose Broth (PDB). Mock was leaves pretreated with water and inoculated with fungus in PDB. Plants were incubated at 24 °C with a 12 h/12 h light/dark cycle (PAR level of 100 μmol m^–2^ s^–1^). Lesion size at 48 h post infection (hpi) was measured using ImageJ software to assess fungal susceptibility. *Pseudomonas syringae* pv *tomato* DC3000 was cultured from a frozen glycerol stock on King Agar B (KB) supplemented with 20 mg/mL proteose peptone, 1.5 mg/mL K_2_HPO_4_, 1.5 mL/mL glycerol, 1.5 mg/mL agarose, 25 μg/mL rifampicin, and 5 mM MgSO_4_. The culture was incubated at 28 °C in the dark for 3 days before inoculum preparation. Four-week-old *Arabidopsis* and five-week-old tomato plants were sprayed with 2 mL of 1 μM flg22 or 2 μg/mL MIPE using adjuvants (0.05% Tween 24 MBAL for *Arabidopsis*; 2.5% Tween 24 MBAL + 2.5% UEP-100 for tomato; Croda, Snaith, UK). Corresponding adjuvant solutions were used as mock. Infection occurred 24 h after pretreatment, as previously described. After 24 h, *Arabidopsis* plants were infected with *P. syringae* pv *tomato* DC3000,^4^ spraying leaves with a bacterial concentration of OD_600_ = 0.1 added with 0.001% of Silwet L-77. Tomato plants were infected, with leaves sprayed with a bacterial concentration of OD_600_ = 0.5 added with 0.002% of Silwet L-77. Leaf discs from both plants were collected at 0 dpi (corresponding to 3 hpi) and 3 dpi to quantify bacterial colonies as cfu per leaf area. Data shown represent mean ± SE (*n* = 6). The experiments were performed three times with similar results.

### 2.10 Analysis of Defense Gene Expression

To evaluate the defense gene expression after MIPE treatment, ten-day-old *Arabidopsis* seedlings were treated for 1 h with flg22 (1 μM), MIPE (2 μg/mL), or water (mock). To assess the MIPE-induced priming effect, *B. cinerea*-infected *Arabidopsis* leaves were collected at 8 hpi. Tissues were homogenized in liquid nitrogen and total RNA was extracted using NucleoZol (Macherey-Nagel, Düren, Germany), treated with DNase (Promega, Southampton, UK), and reverse-transcribed into cDNA. Quantitative reverse transcription PCR (RT-qPCR) was performed with a CFX96 Real-Time System (Bio-Rad, Hercules, CA, USA) using GoTaq qPCR Master Mix (Promega, Southampton, UK). Gene expression was analyzed using the Pfaffl method with *Ubiquitin 5* (*UBQ5*) and *Beta-tubulin 4* (*TUB4*) as reference genes.^3^ Primer sequences were generated with Primer3 software (https://primer3.ut.ee/) (Supporting Information Table S1). Data shown represent mean ± SE (*n* = 3). The experiment was repeated three times with similar results.

### 2.11 Data Analysis

Data were presented as mean ± standard deviation (SD) or standard error (SE) as indicated in the figure legends. The significant differences were evaluated by Student’s *t*-test or ANOVA analysis followed by Tukey’s test (*p* ≤ 0.05), as indicated in the figure legends. Statistical analyses were performed using GraphPad Prism 8.0.1 software (GraphPad Software, San Diego, CA, USA).

## 3 Results

### 3.1 Two-Phase Olive Pomace Digestate Is Enriched in Mineral Nutrients and Characterized by Low Heavy Metal Content

To evaluate the plant fertilization potential of the two-phase olive pomace digestate, biochemical characterization was performed on raw digestate (RD) from a biodigester fed with the two-phase olive pomace. The digestate had a pH of 8.0 ± 0.14, which is suitable for soil compatibility and plant growth. The total solids indicating inorganic and nonvolatile content was 6.69 ± 0.04% (*n* = 3), confirming that it was predominantly aqueous. Organic dry matter was 78.09 ± 0.02% (*n* = 3), indicating that the majority of the dry mass is of organic origin and therefore potentially biodegradable or biologically active. The remaining fraction consisted of inorganic and mineral residues. The C/N ratio of 8.0 ± 0.5 (*n* = 3) reflects a relatively high nitrogen availability compared to carbon, which is consistent with the nitrogen-rich nature of the matrix. Among the macronutrients, potassium (K; 6.8%) was the most abundant, followed by nitrogen (N; 4.9%), ammonium (NH_4_^+^; 2.0%), ammonium nitrogen (NH_4_–N; 1.5%). Minor amounts of phosphorus (P), sulfur (S), magnesium (Mg) (<1% each) were detected (Figure S1A). Micronutrients such as copper (Cu), boron (B), manganese (Mn), zinc (Zn), nickel (Ni) were detected at <0.01% (Figure S1B). Next, we evaluated the potential presence of heavy metals in pomace digestate, which can be present in olives and, consequently, in two-phase olive pomace, as a result of uptake by trees grown in contaminated soil and from foliar applications of fertilizers and pesticides.^65^ Heavy metals, including Cr­(VI) (hexavalent chromium), Hg (mercury), Pb (lead), and Cd (cadmium), were detected only at trace levels (<0.00015%) (Figure S1C). *Salmonella* spp. was not detected, confirming the digestate’s safety as an agricultural amendment. To determine the most plant-beneficial fraction, RD was separated into liquid (LD) and solid (SD) digestates (Figure 1A). The LD comprised 68.5 ± 4.4% of RD (fresh weight), while SD accounted for 32.15 ± 4.3% (w/w). The LD showed similar chemical parameters, nutrient levels, and heavy metal content compared to RD (Figure S1D–F).

Since residual CW-derived polysaccharides in waste biomasses can influence their effectiveness as soil conditioners, they were extracted as alcohol-insoluble solids (AIS) and the monosaccharide composition characterized by high-performance anion-exchange chromatography with pulsed amperometry detection (HPAEC-PAD).^66,67^ LD contained a low monosaccharide content (0.018 ± 0.002%, w/v of LD) indicating a low level of polysaccharides. These were primarily composed of galacturonic acid (∼30%), rhamnose (∼21%), and glucose (∼18%), with minor amounts of arabinose, galactose, mannose, xylose, fucose, and glucuronic acids (Figure S2A). Instead, in SD, the monosaccharide content accounted for 80.2 ± 9.7% w/w SD dry weight, indicating a high content of undigested polysaccharides. The monosaccharide composition of the SD-AIS revealed a high xylose content (∼80%), with lower levels (<10% each) of glucose, galacturonic acid, arabinose, mannose, galactose, and rhamnose, as well as trace amounts of glucuronic acid and fucose (Figure S2B). The high xylose content suggests that SD is rich in hemicelluloses (e.g., xylans) and contains minimal amounts of pectins.

### 3.2 Soil Amendment with Olive Pomace Digestate Positively Influences Plant Growth

The effects of RD as a soil amendment and fertilizer were evaluated in a dose–response experiment with *Arabidopsis* shoot grown in commercial soil, sterilized, and amended with RD at 45, 90, or 180 g/kg. Water-soaked soil was used as a mock. After 2 weeks, rosette fresh and dry weights were measured and compared to the mock (Figure S3A). The 45 g/kg dose increased rosette fresh weight by about 25%, while higher doses (90 and 180 g/kg) resulted in only a 10% increase, with a maximum enhancement of 36%. Similar results were observed for the rosette dry weight (Figure S3B). A dose–response pattern was also observed with tomato, grown in soil amended with RD values of 20, 40, or 80 kg/m^2^. After four months, tomato height was measured, and the 20 kg/m^2^ dose led to a 49% increase in height, with higher doses resulting in smaller increases of around 26%, yielding a total growth increase of 75% (Figure S3C). These findings suggest that higher concentrations reduce the growth response, with 45 and 20 kg/m^2^ being optimal doses for further study in *Arabidopsis* and tomato, respectively.

To compare the biostimulant potential of RD with that of SD and LD, *Arabidopsis* plants were grown in soil amended with RD (45 g/kg), SD (15 g/kg), or LD (30 g/kg). After 2 weeks, rosette fresh and dry weights were measured and compared to a control. All treatments significantly stimulated *Arabidopsis* shoot growth (Figure 1B). RD caused a 43% increase in growth compared to the control, while SD induced a 57% growth increase, and LD exhibited only a 12% increase. Similar results were observed for the rosette dry weight (Figure S3D). The study extended to tomato plants grown in soil amended with an RD (20 kg/m^2^), LD (14 kg/m^2^), or SD (6 kg/m^2^). After four months, growth parameters were quantified and compared to untreated controls (Figure 1D,E). RD amendment significantly increased tomato height and fruit number by 34% and 232%, respectively. Both digestate fractions enhanced tomato height and productivity compared with the control. As seen with *Arabidopsis*, SD produced a more substantial increase in height (63%) and fruit number (315%) than did LD (19% and 150%, respectively). These results indicate that two-phase olive pomace RD can serve as an effective biostimulant for both *Arabidopsis* and tomato, with SD being more effective than LD in stimulating plant growth and fruit production.

### 3.3 Microbial Fraction Reduces Digestate Biostimulant Efficiency

We hypothesized that specific components in the LD fraction might reduce its biostimulatory potential. The presence of galacturonic acid-based carbohydrates in LD (Figure S2A) suggests a possible trade-off between growth and defense responses induced by OG. To explore this hypothesis, pectic fragments were precipitated from LD using ethanol fractionation, and the oligosaccharide profile was analyzed using HPAEC-PAD^4,68^ (Figure S4). However, no OG or other carbohydrate-based elicitor peaks were detected in LD.

The LD fraction contained proteins (1.8 mg/mL). Given that many protein-derived MAMPs are shared across various microbial species and considering that digestates can be enriched with diverse bacterial and fungal populations as well as residual plant biomass, we hypothesized that digestates may function as a continuously proliferating, low-cost reservoir for MAMPs/DAMPs-based phytovaccines. To test this assumption, LD was centrifuged, generating two fractions: a microbial-depleted LD (MD-LD) and a microbial-enriched pellet (M) (Figure 1A). *Arabidopsis* plants were then cultivated in soil amended with varying concentrations of LD, MD-LD (both at 30 g/kg), or M (1.5 g/kg), with M applied at the relative proportion found in LD. After 2 weeks, the rosette fresh and dry weights were measured and compared to the control. Notably, an increase in *Arabidopsis* shoot growth was observed in plants grown in both LD- and MD-LD-amended soil. The increase in rosette fresh weight was significantly higher in the MD-LD treatment compared to the LD treatment, with a difference of approximately 16.7% (Figure 1C). Conversely, the M treatment led to a decrease in plant rosette growth by about 19.2%. Similar results were observed for rosette dry weight (Figure S3E). Plants did not display visible disease symptoms, such as chlorosis, necrosis, or wilting, after M treatment. These data indicate that the microbes present in LD negatively affect its fertilizing potential for both *Arabidopsis* and tomato vegetative growth.

### 3.4 Several Bacterial and Fungal Species Were Identified in the Liquid Fraction of Olive Mill Digestate

Detailed information on the microbial component isolated in the M fraction was obtained through taxonomic characterization of the microbial community. 16S and ITS rRNA metabarcoding was performed to identify bacteria and fungi populations, respectively. The analysis revealed a complex bacterial genus-level community structure, with nearly 50% of sequenced reads assigned to uncultured organisms (Figure 2A). Predominant genera (relative abundance >3%) included *Luteimonas*, *Planomicrobium*, *Caldicoprobacter*, *Pseudomonas*, and *HN-HF0106*. Sequence data indicated also the presence of anaerobic bacteria such as *Tissierella* and *Sedimentibacter* (*Peptostreptococcaceae*) and genera within the *Ruminococcaceae* family (*UCG-010* and *Ruminiclostridium*). Notably, most uncultured organisms were associated with *DTU014* (nearly 40%), followed by *Chloroflexi* (22%), *Firmicutes* (14%), *Collierbacteria* (6%), *SAR324*, and *Bacillaceae* (3%) (Figure 2B). Taxonomic assignment of the fungal community was challenging, likely due to the low DNA concentration in the samples (Figure 2C). This limitation may explain why 71% of the sequences were identified as *Fungi gen. incertae sedis*, indicating undefined broader taxonomic relationships. Despite the low abundance of ITS sequences, notable findings included 7% of the sequences assigned to the genus *Pichia* and *Basidiomycota* and >3% assigned to *Paraglomerales*, *Brettanomyces*, and *Glomus*. No *Salmonella* spp. were detected, indicating the digestate’s potential safety as an agricultural amendment. However, the *Pseudomonas* genera may contain species with pathogenic potential for plants and humans. The genus-level identification performed does not allow definitive exclusion of these pathogenic species, and future studies using species-level metagenomics or culture-based assays will be necessary to fully assess potential risks.

**Figure 2:**
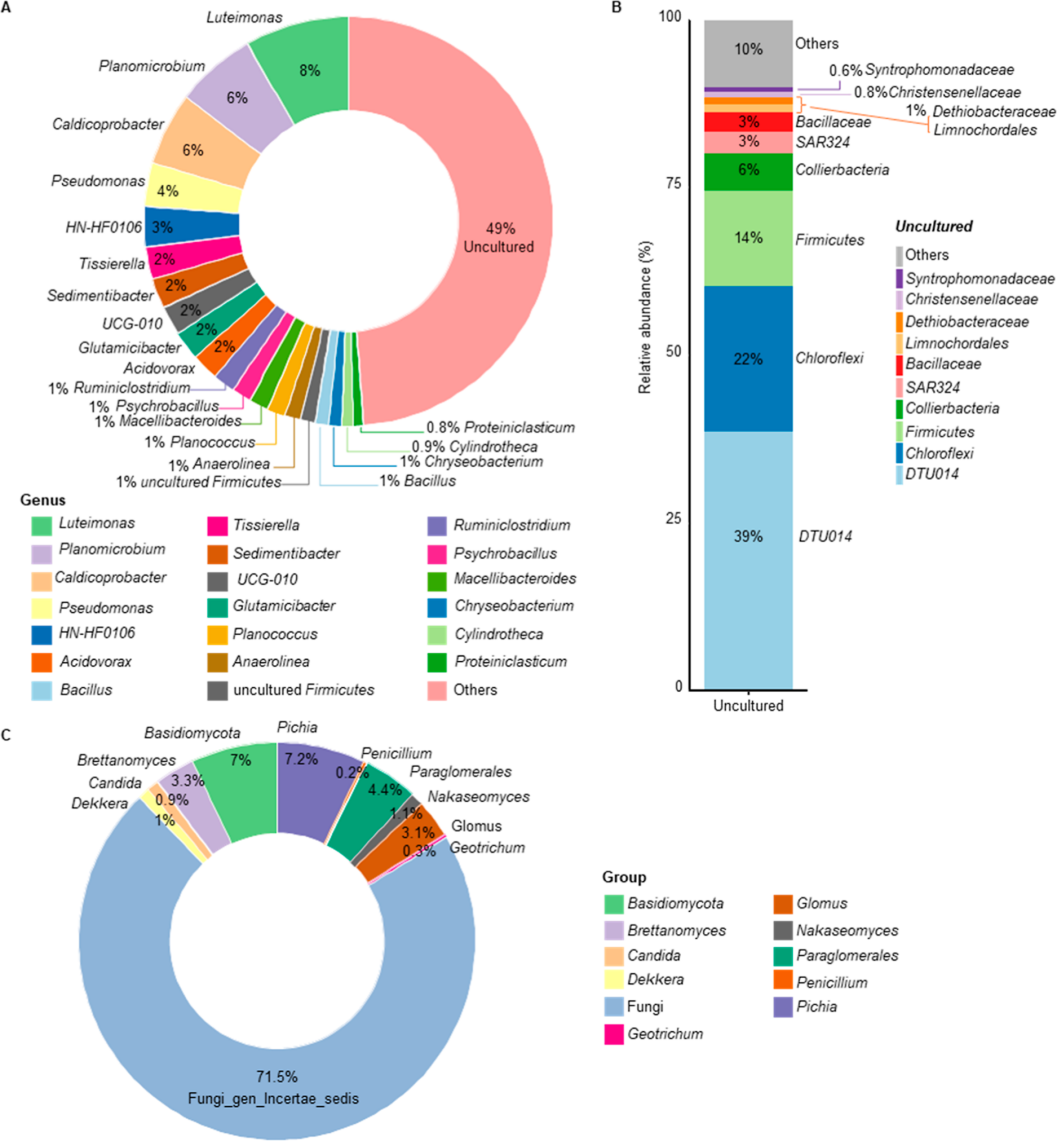
Abundance analysis of 16S rDNA of bacterial cells and rDNA internal transcribed spacer (ITS) region of fungal cells from the digestate. (A) Donut chart describing the proportion of reads assigned to each genus of bacterial community. (B) Bar plot showing the composition of the ≪uncultured≫ fraction of the bacterial community. Each percentage is calculated considering a restricted data set, represented by the hits classified as ≪uncultured≫ at the genus level. (C) Donut chart describing the proportion of reads assigned to each genus of fungal community, represented with different colors. Percentage of abundance is indicated by the integer within each slice. Taxonomic abundances were averaged across replicates (*n* = 10).

### 3.5 Immunogenic Proteins Are Present in Olive Pomace Digestate Fractions

LD was investigated as a potential cost-effective source of MAMPs/DAMPs. A MIPE was obtained from the M fraction of the LD (20 mg powder/L). Proteomic analysis of MIPE by mass spectrometry identified 1169 proteins (Supporting Information Datasheet S2), filtered to 67 consistently detected across biological replicates (Supporting Information Datasheet S3). The identified proteins originated from bacteria (50.7%), fungi (40.3%), and olive plants (8.9%) (Figure S6A). Functional gene ontology (GO) analysis grouped proteins into seven categories, with bacterial proteins linked to host interaction (35.7%), cellular organization (29.2%), metabolism (16.7%), localization (8.3%), and stimulus response (4.2%). Fungal proteins were mainly metabolic (64.3%) or involved in host interactions (21.4%) (Figure S6B). Olive-derived proteins were associated with defense, metabolism, and the cell cycle (33.3% each). The presence of proteins linked to host interaction and defense suggests a role in plant–pathogen interactions. MIPE contained proteins of various sizes, predominantly <300–400 amino acids, indicative of potential elicitor activity, alongside high-molecular-weight proteins (Figure S6C). Intriguingly, microbial functional proteins involved in plant immune responses were detected (Table 1). In particular, precursors of bacterial MAMPs were observed, including elongation factor Tu, releasing a fragment of 18 amino acids (elf18) that can be recognized by Elongation Factor Tu Receptor (EFR) in *Arabidopsis* ^69^ and flagellin, whose immunogenic peptides can be perceived via *Arabidopsis* Flagellin Sensitive 2 (FLS2).^70^ Enzymatic MAMPs derived from bacterial and fungal communities were also detected, including endo-1,4-beta-xylanases A, pectate lyases, and histidine kinases whose elicitor activities can be independent of their enzyme activities.^71–73^ A homologue to golven 1–2A, a phytocytokine known as inducible peptidic DAMP, was also identified.^74,75^ The proteomic analysis also showed plant and microbial enzymes that could produce signaling molecules from hemicellulose components of the plant CW, such as xylanases, and from pectins such as the pectinases rhamnosidases, polygalacturonases, and pectin methylesterases.^32,76–79^

**Table 1:** List of Proteins and Peptides Identified in MIPE, Known to Act as MAMPs, Phytocytokines, or Enzymes That Release DAMPS in Plant Immunity

MAMPs	origin	references
elongation factor Tu	bacteria	^35,69^
flagellin	bacteria	^70,105,106^
endo-1,4-beta-xylanase A	bacteria/fungi	^71^
pectate lyase	bacteria/fungi	^73^
histidine kinase	bacteria/fungi	^72^
Phytocytokines		
homologue to GOLVEN 1–2	olive	^74,75^
Enzymes Potentially Releasing DAMPs		
α-l-rhamnosidase	fungi	^76^
endo-1,4-β-xylanase A	bacteria/fungi	^78^
pectate lyase	bacteria/fungi	^100^

### 3.6 MIPE Induced PTI Hallmarks in *Arabidopsis* and Tomato Plants

We examined whether MIPE can act as an elicitor of plant immune responses. A dose–response analysis was performed on *Arabidopsis* seedling growth with MIPE at 1, 10, or 100 μg dry mass/mL of distilled water (Figure S7A). Seedlings treated with MIPE at 1 and 10 μg/mL showed no significant growth difference from mock-treated seedlings. However, MIPE at 100 μg/mL caused a 35% inhibition in seedling growth. Next, we tested MIPE’s ability to activate PTI hallmarks, such as hydrogen peroxide (H_2_O_2_) production, MAPKs phosphorylation, and upregulation of defense genes. *Arabidopsis* leaf-discs were treated with 1 μg/mL MIPE or with 1 μM of the know elicitor flg22, and H_2_O_2_ production was subsequently quantified. Distilled sterile water was used as mock. The MIPE treatment induced a transient H_2_O_2_ burst (Figure S7B), which was slower than the response to 1 μM flg22 (Figure 3A). Flg22 peaked at 10 min, while MIPE peaked at 20 min. Immunoblot analysis of MAPKs phosphorylation revealed that MIPE induced phosphorylation of MAPK6 and MAPK3 after 5 min, with MAPK4/11 showing slight phosphorylation after 10 min, which increased at 20 min. The phosphorylation pattern of MIPE was similar to flg22, though flg22 induced higher MAPKs phosphorylation earlier (10 min). Next, we explored the expression of three PTI-reporter genes, *CYP81F2*, *FRK1*, and *WRKY53*, after 1 h of *Arabidopsis* seedling treatment with MIPE (1 μg/mL), flg22 (1 μM) or mock (Figure 3C). *CYP81F2* (*CYTOCHROME P450*, *FAMILY 81*) encodes a cytochrome P450 monooxygenase involved in the biosynthesis of indole glucosinolates;^80^ *FRK1* (*FLG22-INDUCED RECEPTOR-LIKE KINASE 1*) encodes a leucine-rich repeat receptor kinase that functions in defense signaling pathways;^81^ *WRKY53* (*WRKY DNA-BINDING PROTEIN 33*) encodes a transcription factor contributing to basal resistance against *P. syringae*.^82^ Both MIPE and flg22 treatments strongly stimulated the expression of all plant immunity genes. Flg22 was more effective in stimulating *CYP81F2* expression, while MIPE induced higher levels of *FRK1* expression. Both MIPE and flg22 treatments showed similar levels of *WRKY53* expression. Overall, our findings suggest that the exogenous application of a specific MIPE concentration to *Arabidopsis* can induce multiple early immune responses. To investigate the effect of MIPE in tomato, leaf discs were treated with 1 μg/mL MIPE, 1 μM flg22, or distilled sterile water as mock, and H_2_O_2_ production was subsequently quantified (Figure 3D). MIPE induced a transient increase in H_2_O_2_ levels (Figure 3E), highlighting its capacity to trigger immune responses in tomato, as also observed for flg22. MIPE reached its maximum at 30 min post-treatment, whereas flg22 induced an earlier peak at 10 min, suggesting that distinct kinetics of signaling activation may be involved.

**Figure 3:**
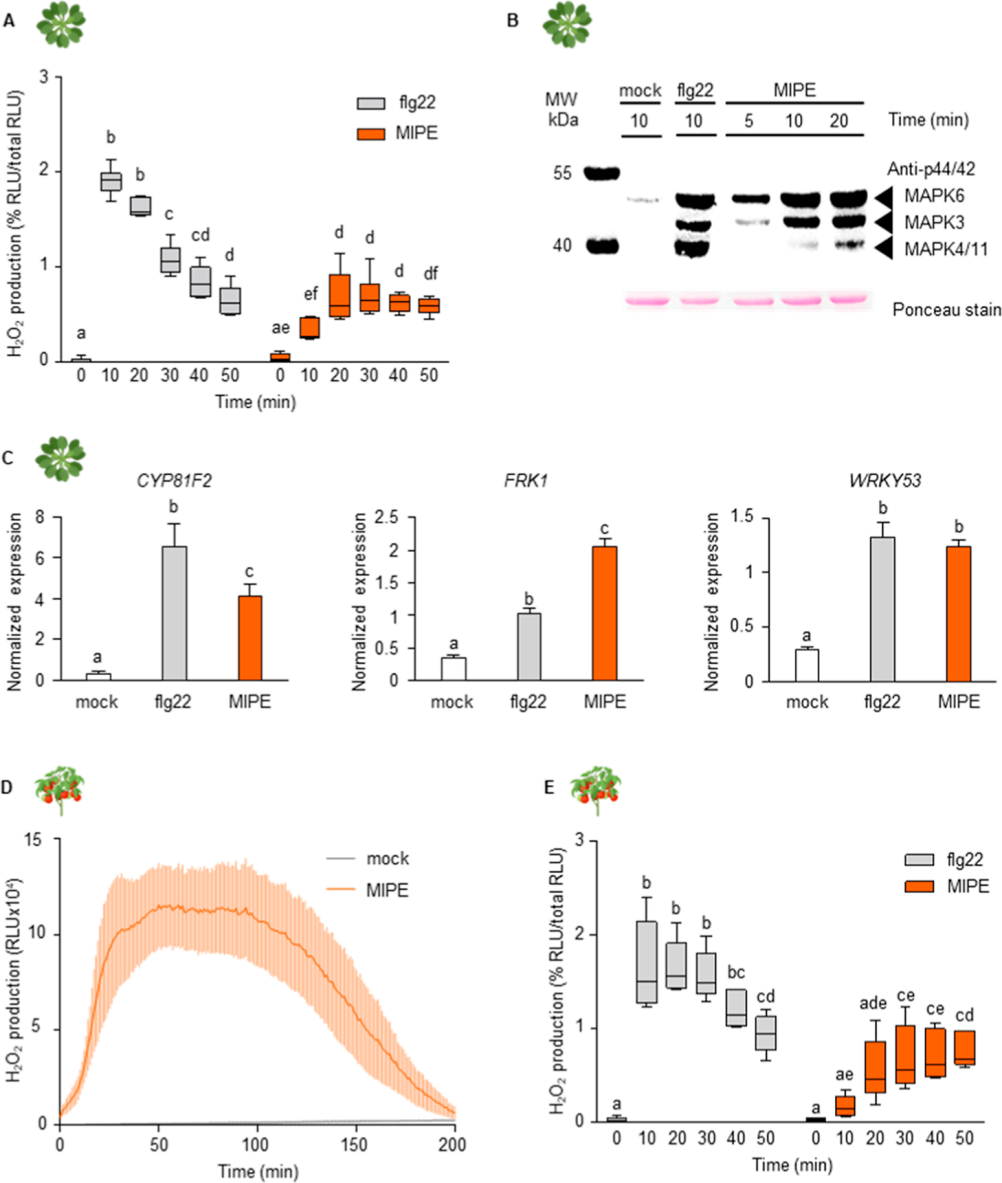
MIPE activated immunity hallmarks in *Arabidopsis* tissues. (A) Measurement of H_2_O_2_ production by luminol reaction after treatment with flg22 (1 μM), or MIPE (1 μg/mL) in four-week-old *Arabidopsis* leaf-discs. The values are reported as ratio of relative luminescence units (RLU) with respect to total RLU at 0, 10, 20, 30, 40, and 50 min. (B) MAPK activation in *Arabidopsis* seedlings in response to sterile distilled water (mock), flg22 (1 μM), or MIPE (1 μg/mL) treatments. MAPKs phosphorylation was determined by Western blot using the phospho-p44/42 MAPKs antibody at different time points (5, 10, and 20 min). Equal protein loading in the gel was confirmed by Ponceau staining. MW = Molecular weight marker. (C) Expression of *CYP81F2, FRK1*, and *WRKY53* after mock, flg22 (1 μM), or MIPE (1 μg/mL) treatments. The expression of defense genes was analyzed by quantitative RT-PCR at 1 h after treatments on 10 days-old *Arabidopsis* seedlings. The expression levels were normalized to *UBQ5* and *TUB4* expression levels. Data represent the mean ± SE (*n* = 3). (D) MIPE activated H_2_O_2_ production in tomato. H_2_O_2_ production measured by luminol reaction for 200 min after treatment with distilled water (mock) or MIPE (1 μg/mL) in five-week-old tomato leaf-discs. Data represent mean ± SE (*n* = 6). (E) Ratio of RLU respect to the total RLU after flg22 (1 μM), or MIPE (1 μg/mL) at 0, 10, 20, 30, 40, and 50 min. Data in A and E are presented as box plots (*n* = 6), with the center line showing the median, the box limits showing the 25th and 75th percentiles, and the whiskers showing the full range of data (minimum to maximum values). Icons next to the graphs indicate the plant species used for the analysis. All the experiments were performed three times with similar results. Different letters indicate significant differences according to ANOVA followed by Tukey’s test (*p* ≤ 0.05).

### 3.7 MIPE Primes Immune Responses and Reduced *Arabidopsis* and Tomato Disease Symptoms Caused by Bacterial and Fungal Pathogens

MIPE’s ability to induce priming responses and protect against *B. cinerea* in *Arabidopsis* was assessed. Four-week-old plants were pretreated with MIPE (1 μg/mL), flg22 (1 μM), or mock, and 24 h later, leaves were inoculated with *B. cinerea* spores (Figure 4A). To test MIPE’s priming effect, the expression of PTI genes *CYP81F2* and *PAD3* (*PHYTOALEXIN DEFICIENT 3*) was evaluated at 8 h post infection (hpi) (Figure 4B). *PAD3* encodes a key biosynthetic enzyme involved in the biosynthesis of the antimicrobial compound camalexin and is essential for elicitor-induced resistance to *B. cinerea*.^83,84^ Both MIPE- and flg22-pretreated plants showed significantly higher expression of *CYP81F2* and *PAD3* compared to mock, with similar induction levels between the two elicitors. At 48 hpi, MIPE and flg22 pretreatments improved *Arabidopsis* resistance to *B. cinerea*, with MIPE-treated plants showing a greater reduction in lesion area (69%) compared to that of flg22 (54%) (Figure 4C). The MIPE induction of *WRKY53* expression previously observed in *Arabidopsis* seedlings suggested a potential priming and protective effect of MIPE against *P. syringae*.^85^ MIPE’s priming effect was also tested in *Arabidopsis* against *P. syringae*. Adult *Arabidopsis* plants were pretreated with MIPE (1 μg/mL), flg22 (1 μM), or mock, and after 24 h, they were inoculated with the bacterium (Figure S8A). The primed state was assessed by monitoring the expression of PTI genes *CYP81F2* and *WRKY53* at 8 hpi (Figure S8B). Both MIPE and flg22 treatments induced a significant upregulation of these genes compared to the mock pretreatment. Typical *Pseudomonas* symptoms, including small necrotic spots with yellow halos, were visible in mock-pretreated plants but absent in pretreated plants (Figure S7C). Bacterial quantification at 0 and 3 days post infection (dpi) (Figure S8C) showed no significant difference at 0 dpi, but at 3 dpi, bacterial growth increased by 39% in mock-pretreated plants, whereas MIPE- and flg22-pretreated plants showed no bacterial growth increase. When pretreated tomato plants were inoculated with *P. syringae* (Figure 4D), bacterial colonies increased by 21% in mock-treated plants at 3 dpi, while MIPE- and flg22-pretreated plants showed no growth increase (Figure 4E). These findings demonstrate that MIPE primes immune responses and protects *Arabidopsis* and tomato against *B. cinerea* and *P. syringae*.

**Figure 4:**
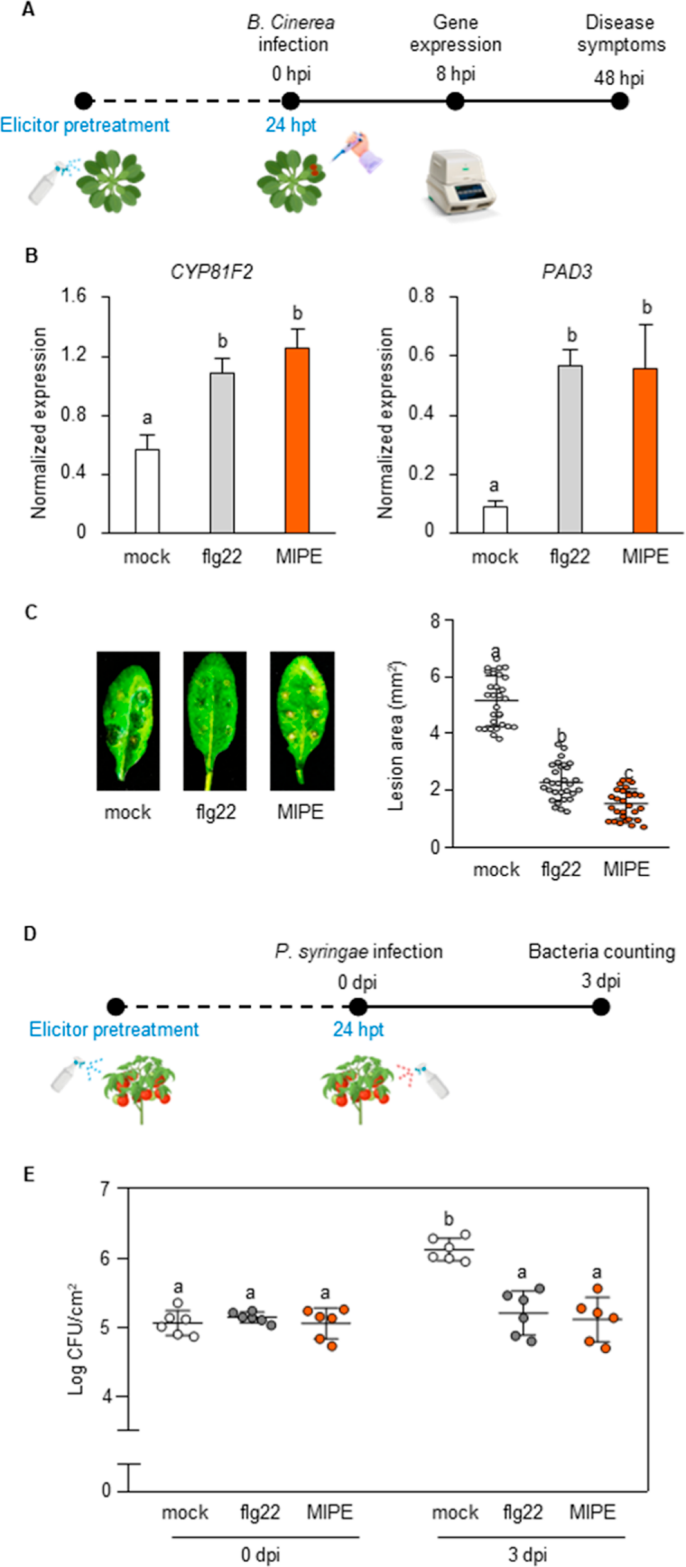
Pretreatment of *Arabidopsis* with MIPE enhanced immune response and protection against *B. cinerea*. (A) Four-week-old *Arabidopsis* leaves were pretreated with to sterile distilled water (mock), flg22 (1 μM), or MIPE (1 μg/mL) and 24 h post pretreatment (hpt) were inoculated with *B. cinerea*. (B) Quantitative RT-PCR analysis of *CYP81F2* and *PAD3* gene expressions in *B. cinerea*-infected leaves collected at 8 h post infection (hpi). mRNA expression levels are normalized to *UBQ5* and *TUB4* expression levels. Data represent the mean ± SE (*n* = 3). (C) Quantification of the lesion areas produced by the spread of *B. cinerea* on *Arabidopsis* leaves at 48 hpi. Values are means ± SD of at least 50 lesions. (D) MIPE induced *P. syringae* pv *tomato* DC3000 resistance in tomato. Leaves of five-week-old tomato Minibel plants were pretreated with adjuvant solutions (mock), flg22 (1 μM), or MIPE (1 μg/mL) 1 day before *P. syringae* inoculation. (E) Bacterial CFU per leaf area (cm^2^) was determined at 0 and 3 days post infection (dpi). Data represent mean ± SD (*n* = 6). All the experiments were performed three times with similar results. Different letters indicate significant differences according to ANOVA followed by Tukey’s test (*p* ≤ 0.05).

## 4 Discussion

This study demonstrates that two-phase olive pomace digestate is a promising biostimulant for plant growth and productivity, extending the findings and discussion presented in a preprint.^86^ The digestate exhibited a pH of 8.0 ± 0.14, which is slightly alkaline and therefore compatible with various soil types, potentially helping to counteract soil acidification, a relevant factor for sustaining plant growth.^87^ The high organic dry matter content emphasizes its richness in organic carbon, contributing to improved soil structure, water retention, and microbial activity, thus positioning it as an excellent soil amendment.^88,89^ The relatively low C/N ratio ensures balanced carbon and nitrogen availability, promoting nutrient cycling and making nitrogen readily accessible to plants.^19^ The high potassium and ammonium content enhances its value as a nutrient-rich fertilizer.^22^ The absence of heavy metals or harmful bacteria, like *Salmonella* spp., ensures safer agricultural use compared to digestates from livestock waste, crop residues, or urban waste, which may contain pollutants and pathogens.^90^ This digestate can be applied directly to the soil as a slurry, either by drenching or irrigation, and may optionally be lightly incorporated into the soil via shallow tillage, depending on agronomic practices.

We highlight the need to refine digestates to enhance its agricultural performance. Separating raw digestate (RD) into solid (SD) and liquid (LD) fractions revealed that SD had a stronger impact on plant growth. SD’s advantage may stem from its higher hemicellulose content, which is more likely to stimulate microbial activity, promote exoenzyme release, and enhance organic matter mineralization.^91^ LD’s lower efficacy was not linked to its mineral content or organic compounds. Our findings indicate that plants grown in soil treated with microbial-depleted liquid digestate (MD-LD) exhibited improved shoot growth compared with those treated with untreated LD, whereas the isolated microbial-enriched pellet (M) caused shoot growth inhibition. Some plant growth-promoting microbes enhance shoot growth over root development to improve plant health and productivity.^92^ Future studies could investigate whether the differences in shoot growth observed following digestate treatment involve a similar mechanism. Many microorganisms present in digestates thrive under anaerobic conditions but are expected to die when the digestate is stored in open ponds or added to soil. This process can trigger the release of MAMPs and DAMPs, which may contribute to the observed reduction in shoot growth through inducing a hyperimmune response, resulting in a growth–defense trade-off.^4,46^

An advanced DNA metabarcoding approach and taxonomic characterization of the microbial pellet revealed a complex and diverse community structure. Notably, nearly 50% of the sequenced reads were associated with uncultured organisms, highlighting the presence of a substantial fraction of unclassified microbial diversity. Predominant genera included *Luteimonas*, *Planomicrobium*, *Caldicoprobacter*, *Pseudomonas*, and *HN-HF0106. Luteimonas*, the most abundant genus in lignocellulosic digestates,^93^ has been associated with plant growth promotion and pathogen resistance.^94^ The olive pomace digestate also hosts a diverse fungal community. Of particular interest was the occurrence of genera *Paraglomerales* and *Glomus*, which belong to the arbuscular mycorrhizal fungi, recognized for enhancing plant growth and health by improving mineral nutrition, as well as by activating defense mechanisms against soilborne pathogens such as *Phytophthora*, *Fusarium*, and *Verticillium*.^95^ Fungal resilience in anaerobic digestion may be due to resistant spores, with post-treatment exposure enhancing colonization and microbial diversity. Not all microorganisms in digestates are strict anaerobes; some can survive under aerobic conditions. Identifying beneficial versus inhibitory microbes is essential for optimizing processing and understanding their impact on soil. Future studies with species-level resolution and functional characterization will be useful to fully evaluate the safety of pomace-derived digestates for agricultural use.

We hypothesized that digestate-derived microbial molecules and plant biomass could be upcycled into bioactive compounds for plant protection. A proteinaceous mixture, MIPE, containing proteins and peptides from bacterial, fungal, and plant sources was isolated. Notably, MIPE includes bacterial and fungal MAMPs precursors like elongation factor Tu, flagellin, endo-1,4-beta-xylanase A, pectate lyase, and histidine kinase, which activate defense responses and confer broad-spectrum pathogen resistance.^96^ MIPE also included the plant GOLVEN 1–2A, reported to regulate root and hypocotyl gravitropism and to act as immunomodulatory phytocytokines.^74^ These peptides are synthesized as inactive precursors and could be processed by plant proteases upon MIPE application, releasing elicitors that activate immune defenses.^75^ The proteomic analysis also revealed a set of CW-degrading enzymes, of both pathogen and plant origin, which can release DAMPs and contribute, together with MAMPs, to plant immune activation during host–pathogen interaction.^97,98^ The causal agent of citrus canker, *Xanthomonas citri* pv *citri* synthesizes xylanases, generating a broad distribution of xyloglucan oligosaccharides.^99^ Pectate lyases could generate OG that are sensed by specific receptors to trigger defense signaling.^100,101^ The other proteins identified within the MIPE fraction may include as yet unknown elicitors, establishing a basis for future scientific investigation.

The EU supports replacing chemical plant protection agents with natural alternatives.^102^ Our findings indicate that MIPE acts as a mix of elicitors initiating PTI. MIPE application to *Arabidopsis* triggered early immune responses including H_2_O_2_ production, MAPKs phosphorylation, and upregulation of PTI-related genes. Our evidence indicates that MIPE can trigger PTI signaling pathways similar to those triggered by canonical elicitors such as flg22 and elf-18.^35,69^ Interestingly, some responses triggered by MIPE, such as *FRK1* expression, MAPK4/11 phosphorylation, and H_2_O_2_ production kinetics, appear to be influenced by the presence of multiple elicitors. The synergistic action of different elicitors in MIPE could lead to a broader coverage of immune responses and increased immune efficiency.^103,104^ Indeed, PTI elicitation by flg22 is enhanced when *Arabidopsis* plants are pretreated with phytocytokine GOLVEN 2 before flg22 application.^74^ Several proteomic-identified proteins with unknown functions could contribute to plant immunity and reveal new bioactive compounds for sustainable agriculture. MIPE pretreatment in *Arabidopsis* primed defense genes (*CYP81F2*, *PAD3*, *WRKY53*) and reduced fungal and bacterial growth. *CYP81F2* activates the indole glucosinolate pathway, producing antimicrobial metabolites that are effective against bacterial and fungal pathogens. *PAD3* drives camalexin biosynthesis, a phytoalexin that restricts fungal colonization, including *B. cinerea*. *WRKY53* functions as a transcription factor coordinating stress-responsive and immune signaling pathways, enabling rapid and robust defense responses and providing broad-spectrum resistance against bacterial and fungal pathogens. The activation of these pathways suggests that MIPE pretreatment stimulates both metabolic and transcriptional layers of the plant immune system, effectively priming both chemical defenses, such as phytoalexins and glucosinolates, and regulatory networks that control stress and immunity. Future studies will be needed to further investigate the dynamics, persistence, and underlying mechanisms of the observed priming. These findings suggest MIPE’s potential as a universal plant immunity elicitor, although further investigation across different plant species is needed. Combining MAMPs, DAMPs, and DAMP-releasing enzymes in MIPE could act as vaccines for plants, enhancing stress memory and climate resilience. Unlike conventional pesticides, which directly target pathogens, MIPE enhances the plant’s own defense mechanisms. Such priming agents could be integrated into crop management strategies to increase resilience to infections, potentially lowering the need for chemical interventions and contributing to more sustainable farming practices. Future studies could explore the long-term effects of MIPE treatment under field conditions and its interactions with other stressors, paving the way for practical applications in agriculture.

In conclusion, this study highlights the significant potential of two-phase olive pomace digestate as a sustainable and versatile agricultural inputs. These findings provide a foundation for developing digestate-based plant biostimulants and immunostimulants across diverse cropping systems and biomass sources. Identification of specific fractions in pomace digestate with differential effects on plant growth and immunity supports targeted upcycling strategies for this biorefinery byproduct. Controlled application of selected fractions after biorefining enhances environmental sustainability by promoting nutrient recycling, reducing reliance on chemical fertilizers and pesticides, and mitigating waste disposal impacts.

## Supplementary Material





## Notes

Proteomic data Including a custom database built combining the UniProtKB reference proteomes; label free MaxQuant data, which contain protein groups resulting from MaxQuant analysis of mass spectrometric RAW files; and list of proteins identified in at least 2 of 3 biological replicates (XLSX)
Additional materials and methods, primers used for quantitative reverse transcript PCR; chemical composition of two-phase olive pomace raw digestate and liquid digestate; characterization of monosaccharide composition in solid and liquid fractions of the two-phase pomace digestate; dose-dependent effects of raw, solid, and liquid digestates on the shoot growth of adult *Arabidopsis* and tomato plants; representation that LD does not contain OGs; MIPE devoid of nucleic acids; taxonomic distribution and functional classification of proteins in MIPE extract; MIPE dose-response on *Arabidopsis* growth and MIPE-induced H_2_O_2_ production; and pre-treatment of Arabidopsis with MIPE enhanced immune response and protection against *P. syringae* (PDF)

## References

[1] Lozano-Castellón J., López-Yerena A., Domínguez-López I., Siscart-Serra A., Fraga N., Sámano S., López-Sabater C., Lamuela-Raventós R. M., Vallverdú-Queralt A., Pérez M. (2022). Extra Virgin Olive Oil: A Comprehensive Review of Efforts
to Ensure Its Authenticity, Traceability, and Safety. Compr. Rev. Food Sci. Food Saf..

[2] Foti P., Pino A., Romeo F. V., Vaccalluzzo A., Caggia C., Randazzo C. L. (2022). Olive Pomace and Pâté
Olive Cake as Suitable Ingredients for Food and Feed. Microorganisms.

[3] Greco M., Fuertes-Rabanal M., Frey C., Grosso C. D., Coculo D., Moretti P., Saldarelli P., Agresti S., Caliandro R., Mélida H., Lionetti V. (2024). Phenolic Compounds-Enriched Extract
Recovered from Two-Phase Olive Pomace Serves as Plant Immunostimulants
and Broad-Spectrum Antimicrobials against Phytopathogens Including *Xylella Fastidiosa*. Plant Stress.

[4] Greco M., Kouzounis D., Fuertes-Rabanal M., Gentile M., Agresti S., Schols H. A., Mélida H., Lionetti V. (2024). Upcycling Olive Pomace
into Pectic Elicitors for Plant Immunity and Disease Protection. Plant Physiol. Biochem..

[5] Donner M., Erraach Y., López-i-Gelats F., Manuel-i-Martin J., Yatribi T., Radić I., El Hadad-Gauthier F. (2022). Circular Bioeconomy
for Olive Oil Waste and By-Product Valorisation: Actors’ Strategies
and Conditions in the Mediterranean Area. J.
Environ. Manage..

[6] Zema D. A., Esteban Lucas-Borja M., Andiloro S., Tamburino V., Zimbone S. M. (2019). Short-Term Effects
of Olive Mill Wastewater Application
on the Hydrological and Physico-Chemical Properties of a Loamy Soil. Agric. Water Manag..

[7] Albalasmeh A. A., Alajlouni M. A., Ghariabeh M. A., Rusan M. J. (2019). Short-Term Effects
of Olive Mill Wastewater Land Spreading on Soil Physical and Hydraulic
Properties. Water Air Soil Pollut..

[8] Atelge M. R., Krisa D., Kumar G., Eskicioglu C., Nguyen D. D., Chang S. W., Atabani A. E., Al-Muhtaseb A. H., Unalan S. (2020). Biogas Production from Organic Waste: Recent Progress
and Perspectives. Waste Biomass Valorization.

[9] Ellacuriaga M., García-Cascallana J., Gómez X. (2021). Biogas Production
from Organic Wastes: Integrating Concepts of Circular Economy. Fuels.

[10] Puig-Gamero M., Trapero J. R., Sánchez P., Sanchez-Silva L. (2020). Is Methanol
Synthesis from Co-Gasification of Olive Pomace and Petcoke Economically
Feasible?. Fuel.

[11] Sánchez-Sánchez C., González-González A., Cuadros-Salcedo F., Cuadros-Blázquez F. (2020). Two-Phase Olive Mill Waste: A Circular
Economy Solution to an Imminent Problem in Southern Europe. J. Clean. Prod..

[12] Polonio D., Gómez-Limón J. A., La Cal J. A., Villanueva A. J. (2024). The Circular
Bioeconomy of the Olive Oil Industry: Deterministic and Probabilistic
Profitability of Olive Mill by-Product Gasification. Biomass Bioenergy.

[13] Blika P. S., Stamatelatou K., Kornaros M., Lyberatos G. (2009). Anaerobic
Digestion of Olive Mill Wastewater. Glob. Nest
J..

[14] Uddin M. A., Siddiki S. Y. A., Ahmed S. F., Rony Z. I., Chowdhury M. A. K., Mofijur M. (2021). Estimation of Sustainable
Bioenergy Production from
Olive Mill Solid Waste. Energies.

[15] Le C., Stuckey D. C. (2017). Impact of Feed Carbohydrates and Nitrogen Source on
the Production of Soluble Microbial Products (SMPs) in Anaerobic Digestion. Water Res..

[16] Santinello D., Zampieri G., Agostini S., Müller B., Favaro L., Treu L., Campanaro S. (2024). Process Stability
in Anaerobic Digestion: Unveiling Microbial Signatures of Full-Scale
Reactor Performance. Chem. Eng. J..

[17] Guan D., Zhao J., Wang Y., Fu Z., Zhang D., Zhang H., Xie J., Sun Y., Zhu J., Wang D. (2024). A Critical Review on Sustainable Management and Resource
Utilization
of Digestate. Process Saf. Environ. Prot..

[18] Carraro G., Tonderski K., Enrich-Prast A. (2024). Solid-Liquid Separation of Digestate
from Biogas Plants: A Systematic Review of the Techniques’
Performance. J. Environ. Manage..

[19] Guilayn F., Jimenez J., Martel J.-L., Rouez M., Crest M., Patureau D. (2019). First Fertilizing-Value Typology
of Digestates: A Decision-Making
Tool for Regulation. Waste Manag..

[20] Guilayn F., Rouez M., Crest M., Patureau D., Jimenez J. (2020). Valorization
of Digestates from Urban or Centralized Biogas Plants: A Critical
Review. Rev. Environ. Sci. Biotechnol..

[21] Möller K. (2015). Effects of
Anaerobic Digestion on Soil Carbon and Nitrogen Turnover, N Emissions,
and Soil Biological Activity. A Review. Agron.
Sustain. Dev..

[22] Weimers K., Bergstrand K.-J., Hultberg M., Asp H. (2022). Liquid Anaerobic Digestate
as Sole Nutrient Source in Soilless Horticulture—Or Spiked
With Mineral Nutrients for Improved Plant Growth. Front. Plant Sci..

[23] Nkoa R. (2014). Agricultural
Benefits and Environmental Risks of Soil Fertilization with Anaerobic
Digestates: A Review. Agron. Sustain. Dev..

[24] Roopnarain A., Akindolire M. A., Rama H., Ndaba B. (2023). Casting Light on the
Micro-Organisms in Digestate: Diversity and Untapped Potential. Fermentation.

[25] Schnürer A., Schnürer J. (2006). Fungal Survival
during Anaerobic Digestion of Organic
Household Waste. Waste Manag..

[26] Zeng J.-Y., Meng M., Qi L., Li Y., Yao H. (2024). Environmental
Risks in Swine Biogas Slurry-Irrigated Soils: A Comprehensive Analysis
of Antibiotic Residues, Resistome, and Bacterial Pathogens. Environ. Int..

[27] Tudi M., Daniel Ruan H., Wang L., Lyu J., Sadler R., Connell D., Chu C., Phung D. T. (2021). Agriculture Development,
Pesticide Application and Its Impact on the Environment. Int. J. Environ. Res. Public Health.

[28] Zipfel C. (2014). Plant Pattern-Recognition
Receptors. Trends Immunol..

[29] Ge D., Yeo I.-C., Shan L. (2022). Knowing Me Knowing You: Self and
Non-Self Recognition in Plant Immunity. Essays
Biochem..

[30] Yuan M., Ngou B. P. M., Ding P., Xin X.-F. (2021). PTI-ETI Crosstalk:
An Integrative View of Plant Immunity. Curr.
Opin. Plant Biol..

[31] Shu L.-J., Kahlon P. S., Ranf S. (2023). The Power
of Patterns: New Insights
into Pattern-Triggered Immunity. New Phytol..

[32] Del
Corpo D., Fullone M. R., Miele R., Lafond M., Pontiggia D., Grisel S., Kieffer-Jaquinod S., Giardina T., Bellincampi D., Lionetti V. (2020). AtPME17 Is a Functional
Arabidopsis Thaliana Pectin Methylesterase Regulated by Its PRO Region
That Triggers PME Activity in the Resistance to Botrytis Cinerea. Mol. Plant Pathol..

[33] Lagrèze J., Pajuelo A. S., Coculo D., Rojas B., Pizzio G. A., Zhang C., Tian M.-B., Malnoy M., Vannozzi A., Costa L. D., Lionetti V., Matus J. T., Malacarne G. (2025). PME10 Is a
Pectin Methylesterase Driving PME Activity and Immunity Against Botrytis
Cinerea in Grapevine (Vitis Vinifera L.). Plant
Biotechnol. J..

[34] Felix G., Duran J. D., Volko S., Boller T. (1999). Plants Have a Sensitive
Perception System for the Most Conserved Domain of Bacterial Flagellin. Plant J..

[35] Kunze G., Zipfel C., Robatzek S., Niehaus K., Boller T., Felix G. (2004). The N Terminus of Bacterial
Elongation Factor Tu Elicits Innate Immunity
in Arabidopsis Plants. Plant Cell.

[36] Klarzynski O., Plesse B., Joubert J.-M., Yvin J.-C., Kopp M., Kloareg B., Fritig B. (2000). Linear β-1,3
Glucans Are Elicitors
of Defense Responses in Tobacco. Plant Physiol..

[37] Kaku H., Nishizawa Y., Ishii-Minami N., Akimoto-Tomiyama C., Dohmae N., Takio K., Minami E., Shibuya N. (2006). Plant Cells
Recognize Chitin Fragments for Defense Signaling through a Plasma
Membrane Receptor. Proc. Natl. Acad. Sci. U.S.A..

[38] Laquitaine L., Gomès E., François J., Marchive C., Pascal S., Hamdi S., Atanassova R., Delrot S., Coutos-Thévenot P. (2006). Molecular
Basis of Ergosterol-Induced Protection of Grape against Botrytis Cinerea:
Induction of Type I LTP Promoter Activity, WRKY, and Stilbene Synthase
Gene Expression. Mol. Plant-Microbe Interact..

[39] Baillieul F., de Ruffray P., Kauffmann S. (2003). Molecular Cloning and Biological
Activity of α-, β-, and γ-Megaspermin, Three Elicitins
Secreted by Phytophthora Megasperma H20. Plant
Physiol..

[40] Gust A. A., Pruitt R., Nürnberger T. (2017). Sensing Danger:
Key to Activating
Plant Immunity. Trends Plant Sci..

[41] Tanaka K., Heil M. (2021). Damage-Associated Molecular
Patterns (DAMPs) in Plant Innate Immunity:
Applying the Danger Model and Evolutionary Perspectives. Annu. Rev. Phytopathol..

[42] Ferrari S., Savatin D. V., Sicilia F., Gramegna G., Cervone F., De Lorenzo G. (2013). Oligogalacturonides:
Plant Damage-Associated Molecular
Patterns and Regulators of Growth and Development. Front. Plant Sci..

[43] Hönig M., Roeber V. M., Schmülling T., Cortleven A. (2023). Chemical Priming
of Plant Defense Responses to Pathogen Attacks. Front. Plant Sci..

[44] Conrath U., Beckers G., Langenbach C., Jaskiewicz M. (2015). Priming for
Enhanced Defense. Annu. Rev. Phytopathol..

[45] Hilker M., Schmülling T. (2019). Stress Priming,
Memory, and Signalling in Plants. Plant Cell
Environ..

[46] He Z., Webster S., He S. Y. (2022). Growth-Defense
Trade-Offs in Plants. Curr. Biol..

[47] Lionetti, V. ; Agresti, S. ; De Lorenzo, G. ; Greco, M. Use of Molecules Isolated from Bacteria Present in Digestate Produced by Biogas Plants as Immunostimulants of Plant Defence Responses. WO 2024188899 A1 IT102023000004800, PCT/EP2024/056272, 2025.

[48] Eddhif B., Lange J., Guignard N., Batonneau Y., Clarhaut J., Papot S., Geffroy-Rodier C., Poinot P. (2018). Study of a Novel Agent for TCA Precipitated Proteins
Washing—Comprehensive Insights into the Role of Ethanol/HCl
on Molten Globule State by Multi-Spectroscopic Analyses. J. Proteomics.

[49] Bradford M. M. (1976). A Rapid
and Sensitive Method for the Quantitation of Microgram Quantities
of Protein Utilizing the Principle of Protein-Dye Binding. Anal. Biochem..

[50] Murashige T., Skoog F. (1962). A Revised Medium for Rapid Growth
and Bio Assays with Tobacco Tissue
Cultures. Physiol. Plant..

[51] Manai M., Fiorillo A., Matuozzo M., Li M., D’Ambrosio C., Franco L., Scaloni A., Fogliano V., Camoni L., Marra M. (2024). Phenotypical and Biochemical
Characterization of Tomato Plants Treated
with Triacontanol. Sci. Rep..

[52] Edwards U., Rogall T., Blöcker H., Emde M., Böttger E. C. (1989). Isolation
and Direct Complete Nucleotide Determination of Entire Genes. Characterization
of a Gene Coding for 16S Ribosomal RNA. Nucleic
Acids Res..

[53] Stackebrandt, E. ; Liesack, W. Nucleic Acids and Classification. In Handbook of New Bacterial Systematics; Academic Press Ltd., 1993; pp 151–194.

[54] Pontiggia D., Spinelli F., Fabbri C., Licursi V., Negri R., De Lorenzo G., Mattei B. (2019). Changes in the Microsomal Proteome
of Tomato Fruit during Ripening. Sci. Rep..

[55] Ciccosanti F., Antonioli M., Sacchi A., Notari S., Farina A., Beccacece A., Fusto M., Vergori A., D’Offizi G., Taglietti F., Antinori A., Nicastri E., Marchioni L., Palmieri F., Ippolito G., Piacentini M., Agrati C., Fimia G. M. (2022). Proteomic Analysis Identifies a Signature
of Disease Severity in the Plasma of COVID-19 Pneumonia Patients Associated
to Neutrophil, Platelet and Complement Activation. Clin. Proteomics.

[56] Tyanova S., Temu T., Carlson A., Sinitcyn P., Mann M., Cox J. (2015). Visualization of LC-MS/MS
Proteomics Data in MaxQuant. Proteomics.

[57] Giovannoni M., Larini I., Scafati V., Scortica A., Compri M., Pontiggia D., Zapparoli G., Vitulo N., Benedetti M., Mattei B. (2021). A Novel Penicillium Sumatraense Isolate Reveals an
Arsenal of Degrading Enzymes Exploitable in Algal Bio-Refinery Processes. Biotechnol. Biofuels.

[58] Okuda S., Yoshizawa A. C., Kobayashi D., Takahashi Y., Watanabe Y., Moriya Y., Hatano A., Takami T., Matsumoto M., Araki N., Tabata T., Iwasaki M., Sugiyama N., Kodera Y., Tanaka S., Goto S., Kawano S., Ishihama Y. (2025). jPOST Environment Accelerates the
Reuse and Reanalysis of Public Proteome Mass Spectrometry Data. Nucleic Acids Res..

[59] IRSA CNR . Quantification of Nitrogen and Ammonium and Related Forms. Quad. 64, 1985. Vol. 3 (Methods n 6–7).

[60] UNI EN 16170:2016 . Sludge, Treated Biowaste and Soil—Determination of Elements Using Inductively Coupled Plasma Optical Emission Spectrometry (ICP-OES); European Committee for Standardization: Brussels. https://store.uni.com/en/uni-en-16170-2016 (accessed 2021–04–10).

[61] UNI EN 16174:2012 . Sludge, Treated Biowaste and Soil—Digestion of Aqua Regia Soluble Fractions of Elements; European Committee for Standardization: Brussels. https://store.uni.com/en/uni-en-16174-2012 (accessed 2021–04–10).

[62] APAT, 3 Man 20/2003 . Metodi microbiologici di analisi del compost, Vol. 3, pp 27–30.

[63] Fuertes-Rabanal M., Largo-Gosens A., Fischer A., Munzert K. S., Carrasco-López C., Sánchez-Vallet A., Engelsdorf T., Mélida H. (2024). Linear β-1,2-Glucans Trigger Immune Hallmarks
and Enhance Disease Resistance in Plants. J.
Exp. Bot..

[64] Lionetti V., Raiola A., Camardella L., Giovane A., Obel N., Pauly M., Favaron F., Cervone F., Bellincampi D. (2007). Overexpression
of Pectin Methylesterase Inhibitors in Arabidopsis Restricts Fungal
Infection by Botrytis Cinerea. Plant Physiol..

[65] Charfi A., Moya López A. J., Sánchez Villasclaras S. (2025). Food Safety
in the Production of Olive Oils. Presence of Heavy Metals and Phthalic
Acid Esters Using Different Types of Packaging. J. Food Sci. Technol..

[66] Izydorczyk G., Skrzypczak D., Mironiuk M., Mikula K., Samoraj M., Gil F., Taf R., Moustakas K., Chojnacka K. (2024). Lignocellulosic
Biomass Fertilizers: Production, Characterization, and Agri-Applications. Sci. Total Environ..

[67] Lionetti V., Fabri E., De Caroli M., Hansen A. R., Willats W. G. T., Piro G., Bellincampi D. (2017). Three Pectin
Methylesterase Inhibitors
Protect Cell Wall Integrity for Arabidopsis Immunity to Botrytis. Plant Physiol..

[68] Lionetti V. (2015). PECTOPLATE:
The Simultaneous Phenotyping of Pectin Methylesterases, Pectinases,
and Oligogalacturonides in Plants during Biotic Stresses. Front. Plant Sci..

[69] Zipfel C., Kunze G., Chinchilla D., Caniard A., Jones J. D. G., Boller T., Felix G. (2006). Perception
of the Bacterial PAMP
EF-Tu by the Receptor EFR Restricts Agrobacterium-Mediated Transformation. Cell.

[70] Zipfel C., Robatzek S., Navarro L., Oakeley E. J., Jones J. D. G., Felix G., Boller T. (2004). Bacterial
Disease Resistance in Arabidopsis
through Flagellin Perception. Nature.

[71] Ron M., Avni A. (2004). The Receptor for the
Fungal Elicitor Ethylene-Inducing Xylanase Is
a Member of a Resistance-Like Gene Family in Tomato. Plant Cell.

[72] Seo S., Nakaho K., Hong S. W., Takahashi H., Shigemori H., Mitsuhara I. (2016). L-Histidine Induces Resistance in
Plants to the Bacterial Pathogen Ralstonia Solanacearum Partially
Through the Activation of Ethylene Signaling. Plant Cell Physiol..

[73] Wang C., Huang Z., Duan Z., Zhu L., Di R., Bao Y., Powell C. A., Hu Q., Chen B., Zhang M., Yao W. (2023). Pectate Lyase from Fusarium Sacchari Induces Plant Immune Responses
and Contributes to Virulence. Microbiol. Spectr..

[74] Stegmann M., Zecua-Ramirez P., Ludwig C., Lee H.-S., Peterson B., Nimchuk Z. L., Belkhadir Y., Hückelhoven R. (2022). RGI-GOLVEN
Signaling Promotes Cell Surface Immune Receptor Abundance to Regulate
Plant Immunity. EMBO Rep..

[75] Del
Corpo D., Coculo D., Greco M., De Lorenzo G., Lionetti V. (2024). Pull the Fuzes: Processing Protein Precursors to Generate
Apoplastic Danger Signals for Triggering Plant Immunity. Plant Commun..

[76] Jiménez-Maldonado M. I., Tiznado-Hernández M. E., Rascón-Chu A., Carvajal-Millan E., Lizardi-Mendoza J., Troncoso-Rojas R. (2018). Analysis of
Rhamnogalacturonan I Fragments as Elicitors of the Defense Mechanism
in Tomato Fruit. Chil. J. Agric. Res..

[77] Yang Y., Zhang Y., Li B., Yang X., Dong Y., Qiu D. (2018). A Verticillium Dahliae
Pectate Lyase Induces Plant Immune Responses
and Contributes to Virulence. Front. Plant Sci..

[78] Mélida H., Bacete L., Ruprecht C., Rebaque D., del Hierro I., López G., Brunner F., Pfrengle F., Molina A. (2020). Arabinoxylan-Oligosaccharides
Act as Damage Associated Molecular Patterns in Plants Regulating Disease
Resistance. Front. Plant Sci..

[79] Coculo D., Del Corpo D., Martínez M. O., Vera P., Piro G., De Caroli M., Lionetti V. (2023). Arabidopsis Subtilases Promote Defense-Related
Pectin Methylesterase Activity and Robust Immune Responses to Botrytis
Infection. Plant Physiol. Biochem..

[80] Pfalz M., Vogel H., Kroymann J. (2009). The Gene Controlling
the Indole Glucosinolate
Modifier1 Quantitative Trait Locus Alters Indole Glucosinolate Structures
and Aphid Resistance in Arabidopsis. Plant Cell.

[81] Asai T., Tena G., Plotnikova J., Willmann M. R., Chiu W. L., Gomez-Gomez L., Boller T., Ausubel F. M., Sheen J. (2002). MAP Kinase
Signalling Cascade in Arabidopsis Innate Immunity. Nature.

[82] Murray S. L., Ingle R. A., Petersen L. N., Denby K. J. (2007). Basal Resistance
Against Pseudomonas Syringae in Arabidopsis Involves WRKY53 and a
Protein with Homology to a Nematode Resistance Protein. Mol. Plant. Microbe Interact..

[83] Ferrari S., Galletti R., Denoux C., De Lorenzo G., Ausubel F. M., Dewdney J. (2007). Resistance to Botrytis Cinerea Induced
in Arabidopsis by Elicitors Is Independent of Salicylic Acid, Ethylene,
or Jasmonate Signaling but Requires PHYTOALEXIN DEFICIENT3. Plant Physiol..

[84] Schuhegger R., Nafisi M., Mansourova M., Petersen B. L., Olsen C. E., Svatoš A., Halkier B. A., Glawischnig E. (2006). CYP71B15 (PAD3)
Catalyzes the Final Step in Camalexin Biosynthesis. Plant Physiol..

[85] Hu Y., Dong Q., Yu D. (2012). Arabidopsis WRKY46 Coordinates with
WRKY70 and WRKY53 in Basal Resistance against Pathogen Pseudomonas
Syringae. Plant Sci..

[86] Greco M., Coculo D., Conti A., Abatematteo M., Agresti S., Pontiggia D., Mélida H., Favaro L., Lionetti V. (2025). Enhancing Plant Immune
Training and
Protection through Damage- and Microbe-Associated Molecular Patterns
from Anaerobic Digestate. bioRxiv.

[87] Lencioni G., Imperiale D., Cavirani N., Marmiroli N., Marmiroli M. (2016). Environmental Application and Phytotoxicity of Anaerobic
Digestate from Pig Farming by in Vitro and in Vivo Trials. Int. J. Environ. Sci. Technol..

[88] Li F., Yuan Y., Gong P., Imazumi Y., Na R., Shimizu N. (2023). Comparative Effects of Mineral Fertilizer and Digestate
on Growth, Antioxidant System, and Physiology of Lettuce under Salt
Stress. Hortic., Environ. Biotechnol..

[89] Mayerová M., Šimon T., Stehlík M., Madaras M., Koubová M., Smatanová M. (2023). Long-Term Application of Biogas Digestate Improves
Soil Physical Properties. Soil Tillage Res..

[90] Siebielec G., Siebielec S., Lipski D. (2018). Long-Term Impact of
Sewage Sludge,
Digestate and Mineral Fertilizers on Plant Yield and Soil Biological
Activity. J. Clean. Prod..

[91] Hicks L. C., Meir P., Nottingham A. T., Reay D. S., Stott A. W., Salinas N., Whitaker J. (2019). Carbon and
Nitrogen Inputs Differentially
Affect Priming of Soil Organic Matter in Tropical Lowland and Montane
Soils. Soil Biol. Biochem..

[92] Verbon E. H., Liberman L. M. (2016). Beneficial Microbes Affect Endogenous
Mechanisms Controlling
Root Development. Trends Plant Sci..

[93] Akyol Ç., Ince O., Ince B. (2019). Crop-Based
Composting of Lignocellulosic
Digestates: Focus on Bacterial and Fungal Diversity. Bioresour. Technol..

[94] Ulrich K., Becker R., Behrendt U., Kube M., Schneck V., Ulrich A. (2022). Physiological and Genomic
Characterisation of *Luteimonas Fraxinea* Sp. Nov.,
a Bacterial Species Associated
with Trees Tolerant to Ash Dieback. Syst. Appl.
Microbiol..

[95] Thenappan D. P., Thompson D., Joshi M., Mishra A. K., Joshi V. (2024). Unraveling
the Spatio-Temporal Dynamics of Soil and Root-Associated Microbiomes
in Texas Olive Orchards. Sci. Rep..

[96] Dodds P. N., Chen J., Outram M. A. (2024). Pathogen Perception
and Signaling
in Plant Immunity. Plant Cell.

[97] Bellincampi D., Cervone F., Lionetti V. (2014). Plant Cell
Wall Dynamics and Wall-Related
Susceptibility in Plant-Pathogen Interactions. Front. Plant Sci..

[98] Swaminathan S., Lionetti V., Zabotina O. A. (2022). Plant Cell
Wall Integrity Perturbations
and Priming for Defense. Plants.

[99] Vieira P. S., Bonfim I. M., Araujo E. A., Melo R. R., Lima A. R., Fessel M. R., Paixão D. A. A., Persinoti G. F., Rocco S. A., Lima T. B., Pirolla R. A. S., Morais M. A. B., Correa J. B. L., Zanphorlin L. M., Diogo J. A., Lima E. A., Grandis A., Buckeridge M. S., Gozzo F. C., Benedetti C. E., Polikarpov I., Giuseppe P. O., Murakami M. T. (2021). Xyloglucan Processing
Machinery in Xanthomonas Pathogens and Its Role in the Transcriptional
Activation of Virulence Factors. Nat. Commun..

[100] Osorio S., Castillejo C., Quesada M. A., Medina-Escobar N., Brownsey G. J., Suau R., Heredia A., Botella M. A., Valpuesta V. (2008). Partial Demethylation of Oligogalacturonides by Pectin
Methyl Esterase 1 Is Required for Eliciting Defence Responses in Wild
Strawberry (Fragaria Vesca). Plant J. Cell Mol.
Biol..

[101] Lionetti V., Cervone F., Bellincampi D. (2012). Methyl Esterification
of Pectin Plays a Role during Plant–Pathogen Interactions and
Affects Plant Resistance to Diseases. J. Plant
Physiol..

[102] Drobek M., Frąc M., Cybulska J. (2019). Plant Biostimulants:
Importance of the Quality and Yield of Horticultural Crops and the
Improvement of Plant Tolerance to Abiotic Stress—A Review. Agronomy.

[103] Tundo S., Kalunke R., Janni M., Volpi C., Lionetti V., Bellincampi D., Favaron F., D’Ovidio R. (2016). Pyramiding
PvPGIP2 and TAXI-III But Not PvPGIP2 and PMEI Enhances Resistance
Against Fusarium Graminearum. Mol. Plant-Microbe
Interact..

[104] Swaminathan S., Reem N. T., Lionetti V., Zabotina O. A. (2021). Coexpression
of Fungal Cell Wall-Modifying Enzymes Reveals Their Additive Impact
on Arabidopsis Resistance to the Fungal Pathogen, Botrytis Cinerea. Biology.

[105] Gómez-Gómez L., Boller T. (2000). FLS2: An LRR
Receptor-like
Kinase Involved in the Perception of the Bacterial Elicitor Flagellin
in Arabidopsis. Mol. Cell.

[106] Denoux C., Galletti R., Mammarella N., Gopalan S., Werck D., De Lorenzo G., Ferrari S., Ausubel F. M., Dewdney J. (2008). Activation of Defense
Response Pathways by OGs and Flg22 Elicitors in Arabidopsis Seedlings. Mol. Plant.

