# Supplementary material for: Biorefining of Anaerobic Digestates for the Recovery of Biostimulants and Bioelicitors for Immune Priming and Plant Protection
Source: Environ Sci Technol. 2025 Sep 30;59(40):21700–14. doi: 10.1021/acs.est.5c03321 (PMC12529957; doi:10.1021/acs.est.5c03321)
Supplement: Supplementary file 2 [file es5c03321_si_002.pdf]

Supporting Information for

Biorefining of anaerobic digestate for the recovery of biostimulants and bioelicitors for immune priming and plant protection

Marco Greco[1], Daniele Coculo[1], Angela Conti[2], Savino Agresti[3], Daniela Pontiggia[1,4], Hugo Mélida[5,6], Lorenzo Favaro[7,8], and Vincenzo Lionetti[1,4*]

[1]Department of Biology and Biotechnologies "Charles Darwin", Sapienza University of Rome, Rome, Italy

[2]Department of Pharmaceutical Sciences, University of Perugia, Perugia, Italy

[3]Agrolio s.r.l., S.P. 231 KM 55+120, 70031, Andria, Puglia, Italy

[4]Research Center for Applied Sciences to the Safeguard of Environment and Cultural Heritage (CIABC), Sapienza University of Rome, Rome, Italy

[5]Área de Fisiología Vegetal, Departamento de Ingeniería y Ciencias Agrarias, Universidad de León, León, Spain

[6]Instituto de Biología Molecular, Genómica y Proteómica (INBIOMIC), Universidad de León, León, Spain

[7]Department of Agronomy Food Natural resources Animals and Environment (DAFNAE), University of Padova, Agripolis, Legnaro, PD, Italy

[8]Department of Microbiology, Stellenbosch University, Private Bag X1, Matieland 7602, South Africa

*Corresponding author: Vincenzo Lionetti
vincenzo.lionetti@uniroma1.it

Number of pages 11
Number of text 1
Number of tables 1
Number of figures 8

Text S1 Characterization of Monosaccharide Composition and oligosaccharide content in digestate
LD and SD fractions were lyophilized, and carbohydrates were extracted as alcohol-insoluble solids (AIS) [1]. Two mg of AIS were hydrolyzed in 2 M trifluoroacetic acid at 121°C. Monosaccharide composition of TFA-hydrolyzed AIS was determined by high-performance anion-exchange chromatography with pulsed amperometric detection (HPAEC-PAD; PA20 column, Dionex) and quantified using a standard mixture of the different monosaccharides Fucose (Fuc), Rhamnose (Rha), Arabinose (Ara), Galactose (Gal), Glucose (Glc), Xylose (Xyl), Mannose (Man), Galacturonic Acid (GalA), and Glucuronic Acid (GlcA) (Sigma-Aldrich). Data represent the mean ± standard deviation ($n \geq 3$). The different letters above the boxplots indicate significantly different datasets according to ANOVA followed by Tukey's test ($p \leq 0.05$). Oligosaccharide characterization in LD was performed as previously reported [2,3]. Specifically, the isolation of oligosaccharides from LD was carried out by adding 50 mM acetic acid to reach pH 5, and 11% EtOH (v/v) from cold 100% EtOH solvent while stirring on ice [4]. Then, the samples were incubated overnight at 4 °C with constant orbital shaking (50 rpm) and then centrifuged at 30000×g for 30 min at 4 °C. The resulting supernatant was discarded and the pellet (alcohol insoluble solids; AIS) was collected for subsequent steps. AIS was re-dissolved at 1 g/ml in water, dialyzed against ultrapure water in a dialysis tube with a molecular weight cut-off (MWCO) of 1000 Da (Spectra/Por®, 6 Dialysis Membrane, part number: 132636), and dried. The oligosaccharide profile of LD was investigated by HPAEC-PAD with an ICS-3000 apparatus (Dionex Corporation, Sunnyvale, CA, USA) equipped with a CarboPac PA-200 separation column (2 mm ID × 250 mm; Dionex Corporation) and a Carbopac PA-200 guard column (2 mm ID × 50 mm; Dionex Corporation). A flow of 0.4 mL/min was used and the temperature was kept at 25°C. The injected samples (25 µL) were separated using a linear gradient of 0.05 M KOH (solvent A) and 1 M KOAc in 0.05 M KOH (solvent B) using the following conditions: 0–31 min from 90% A to 20% A and from 10% B to 80% B. Before injection of each sample, the column was equilibrated with 90% A and 10% B for 10 min. Peaks were compared with Oligogalacturonides (OGs) having known Degree of Polymarization (DPs). The experiments were performed three times with similar results.

Reference of supplementary texts

(1) Lionetti, V.; Fabri, E.; De Caroli, M.; Hansen, A. R.; Willats, W. G. T.; Piro, G.; Bellincampi, D. Three Pectin Methylesterase Inhibitors Protect Cell Wall Integrity for Arabidopsis Immunity to Botrytis. *Plant Physiol.* **2017**, *173* (3), 1844–1863. https://doi.org/10.1104/pp.16.01185.

(2) Greco, M.; Kouzounis, D.; Fuertes-Rabanal, M.; Gentile, M.; Agresti, S.; Schols, H. A.; Mélida, H.; Lionetti, V. Upcycling Olive Pomace into Pectic Elicitors for Plant Immunity and Disease Protection. *Plant Physiol. Biochem.* **2024**, *217*, 109213. https://doi.org/10.1016/j.plaphy.2024.109213.

(3) Lionetti, V. PECTOPLATE: The Simultaneous Phenotyping of Pectin Methylesterases, Pectinases, and Oligogalacturonides in Plants during Biotic Stresses. *Front. Plant Sci.* **2015**, *6*.

(4) Spiro, M. D.; Kates, K. A.; Koller, A. L.; O'Neill, M. A.; Albersheim, P.; Darvill, A. G. Purification and Characterization of Biologically Active 1,4-Linked α-d-Oligogalacturonides after Partial Digestion of Polygalacturonic Acid with Endopolygalacturonase. *Carbohydr. Res.* **1993**, *247*, 9–20. https://doi.org/10.1016/0008-6215(93)84237-Z.

Table S1. Primers used for Quantitative Reverse Transcript PCR.

GENE	AGI CODE	FORWARD PRIMER (5'-3')	REVERSE PRIMER (5'-3')
FRK1	AT2G19190	GCTTGTACCGAGCACACTTCTG	CAGTCACTATGCCATAAACAATCTGTT
WRKY53	AT4G23810	CACCAGAGTCAAACCAGCCATTA	CTTTACCATCATCAAGCCCATCGG
CYP81F2	AT5G57220	GTGAAAGCACTAGGCGAAGC	ATCCGTTCCAGCTAGCATCA
UBQ5	AT5G25760	CCGTGGTGGTGCTAAGAAGA	AGCTCCACAGGTTGCGTTAG
PAD3	AT3G26830	TCGCTGGCATAACACTATGG	TTGGGAGCAAGAGTGGAGT
TUB4	AT5G44340	AACGCTGACGAGTGTATGGTT	CCAAAGGTAGGATTAGCGAGC

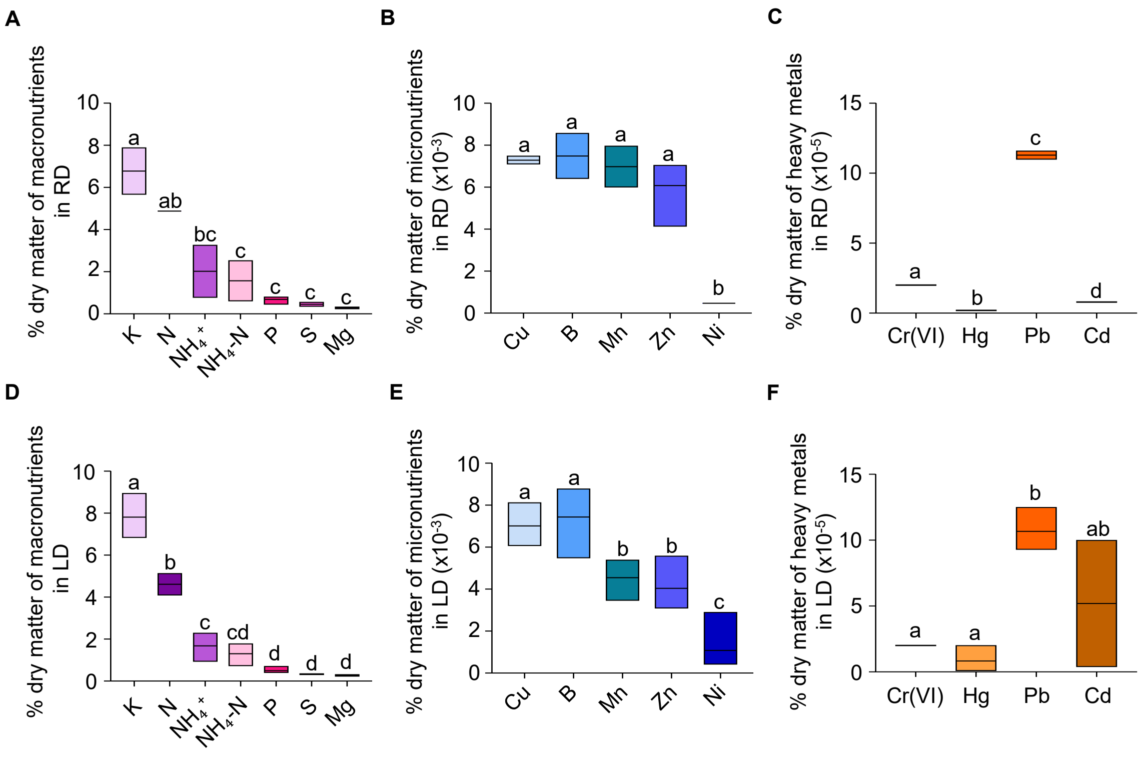

Figure S1. Representation of chemical composition of the two-phase olive pomace raw digestate and liquid digestate (A-B-C) Distribution of macro- and micro-nutrients, and heavy metals in raw digestate (RD). The values are expressed as % dry matter/RD dry matter. (**D-E-F**) Distribution of macro- and micro-nutrients, and heavy metals in liquid digestate (LD). The values are expressed as % dry matter/LD. Data represent the mean ± SD ($n \geq 3$). The different letters indicate significantly different datasets according to ANOVA followed by Tukey's test ($p \leq 0.05$). Potassium (K), Nitrogen (N), Ammonium (NH_4^+), Ammonium nitrogen (NH_4-N), Phosphorus (P), Sulfur (S), Magnesium (Mg), Copper (Cu), Boron (B), Manganese (Mn), Zinc (Zn), Nickel (Ni).

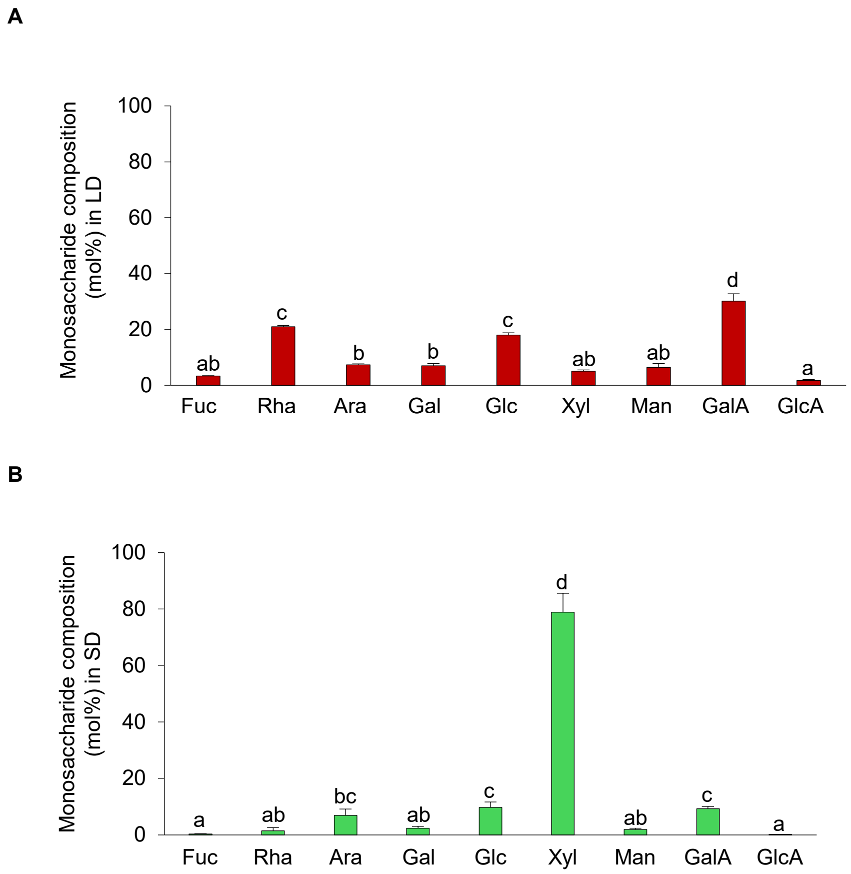

Figure S2. Characterization of monosaccharide composition in solid and liquid fractions of the two-phase pomace digestate. Monosaccharide compositions of liquid digestate (LD) (A) and solid digestate (SD) (B). The molar percentages (mol %) of Fucose (Fuc), Rhamnose (Rha), Arabinose (Ara), Galactose (Gal), Glucose (Glc), Xylose (Xyl), Mannose (Man), Galacturonic Acid (GalA), and Glucuronic Acid (GlcA) were quantified. Results represent the mean ± SD (n=3). The different letters indicate significantly different datasets according to ANOVA followed by Tukey's test ($p < 0.05$).

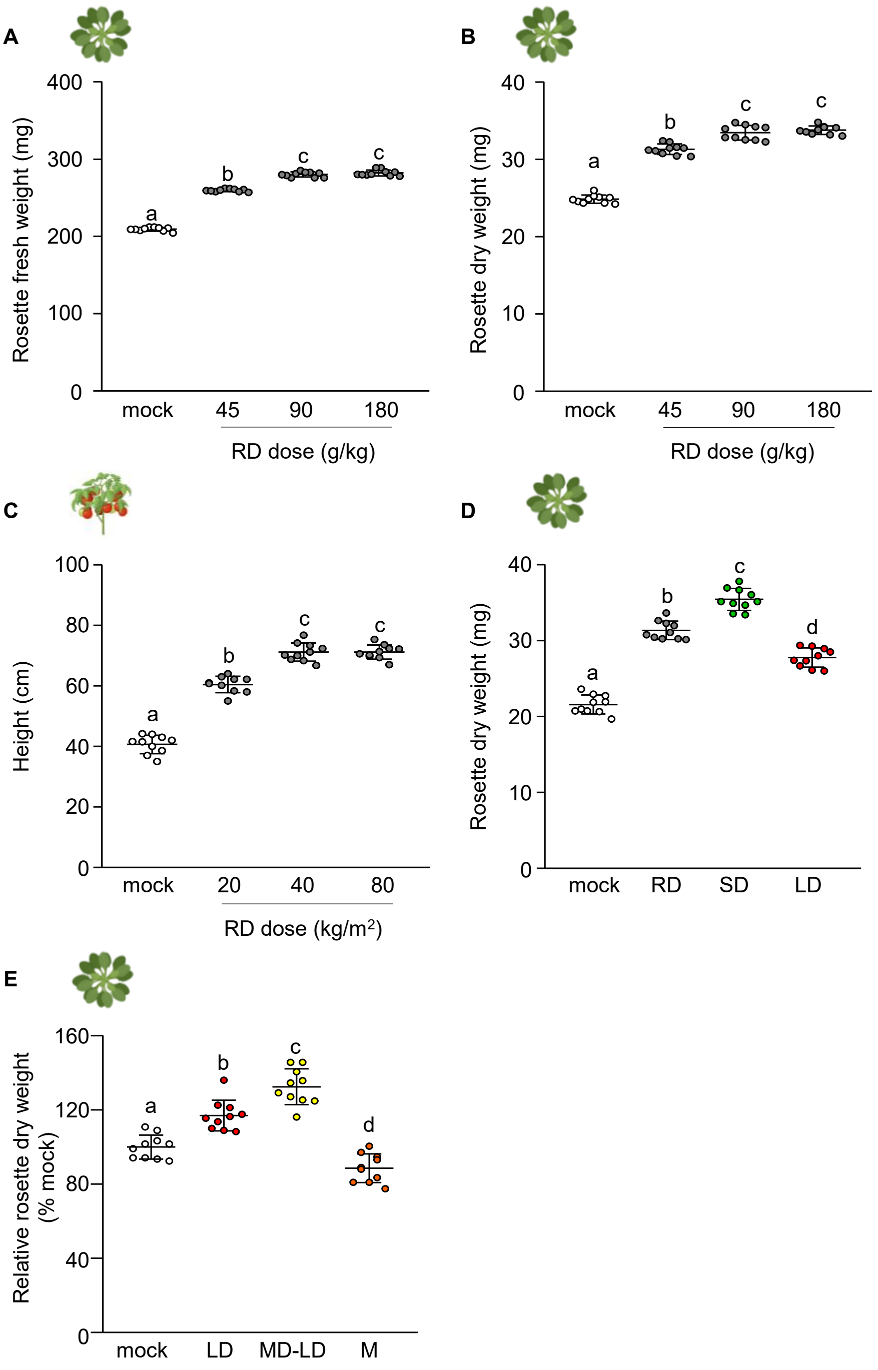

Figure S3. Dose-dependent effects of raw, solid, and liquid digestate on the shoot growth of adult Arabidopsis and tomato plants (**A-B**) Dose-response effects of soil amended with RD at different doses (45, 90, or 180 g/kg) and mock on *A. thaliana* shoot growth (rosette fresh weight, A; and dry weight, B). (**C**) Dose-effects of soil amended with RD (20, 40, or 80 kg/m^2) on height of tomato grown on field. (**D**) Effects of soil amended with RD (45 g/kg), SD (15 g/kg), or LD (30 g/kg) fractions on Arabidopsis shoot growth, measured as rosette dry weight. (**E**) Effects of soil amended with LD, MD-LD (both 30 g/kg), or M (1.5 g/kg) on Arabidopsis shoot growth, measured as relative rosette dry weight compared to mock. The values are expressed as percentages relative to plant grown on water-soaked soil used as mock. Icons next to the graphs indicate the plant species used for the analysis. Data shown represent the mean ±SD (n=10). The experiments were repeated three times with similar results. The different letters indicate significantly different datasets according to ANOVA followed by Tukey's test ($p \leq 0.05$).

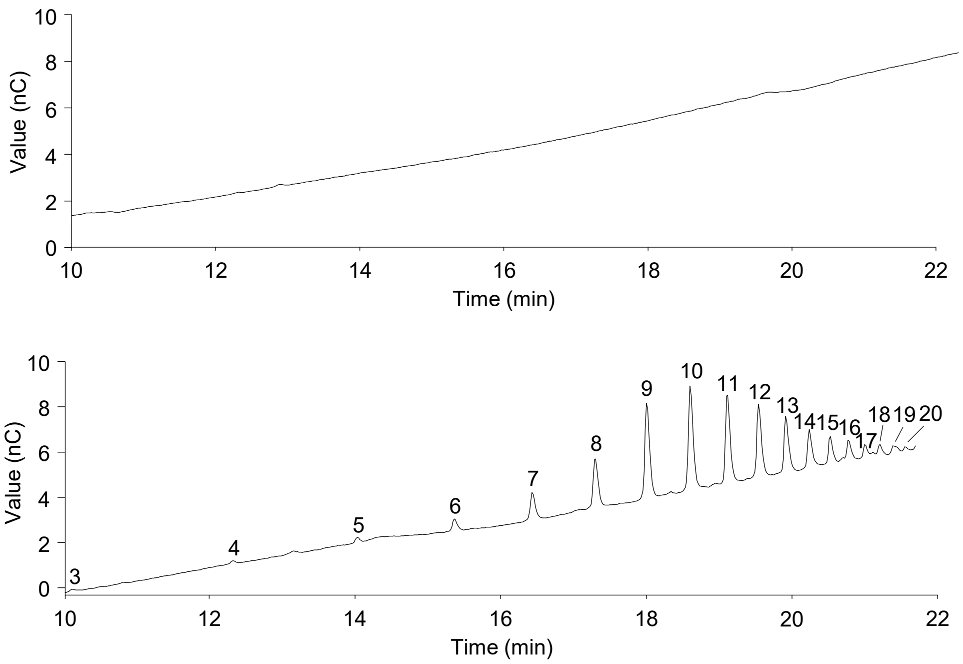

Figure S4. LD does not contain oligogalacturonides (OGs). HPAEC-PAD profiles of OG in LD (upper panel). A mixture of OG 5-20 was used as standard. Numbers indicate the DP of each OG peak. The chromatogram shows the intensity of the signals (nC) plotted against retention time (min).

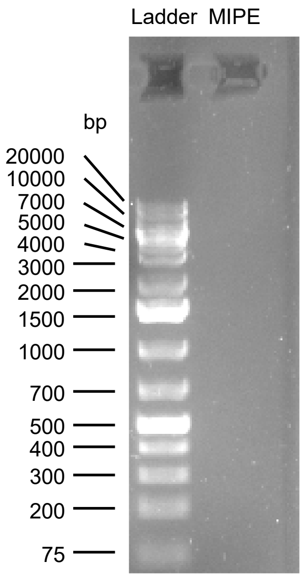

Figure S5. MIPE is devoid of nucleic acids. Electrophoresis on a 1.5% (w/v) agarose gel carried out at 60 V for 25 min. Images were captured using Gel Doc™ XR + System (BioRad). Bands indicate nucleic acids at different lengths measured in base pairs (bp).

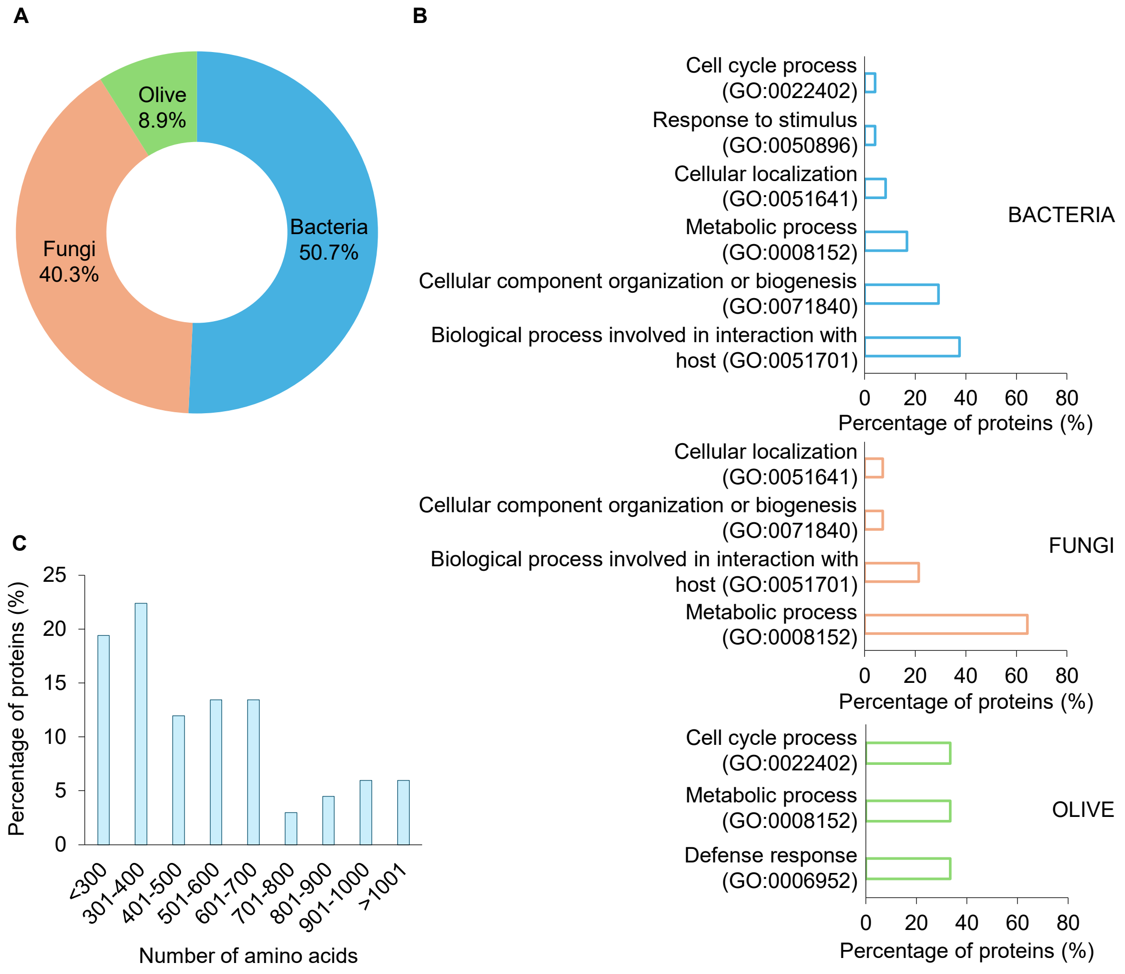

Figure S6. Taxonomic distribution and functional classification of proteins in MIPE extract (**A**) Donut chart describing the proportion of protein assigned to bacteria, fungi or olive, represented with different colors. Percentage of abundance is indicated by the integer within each slice. (**B**) GO term category of biological processes of identified proteins sorted by bacteria, fungi, and olive groups. (**C**) Amino acids length of identified proteins.

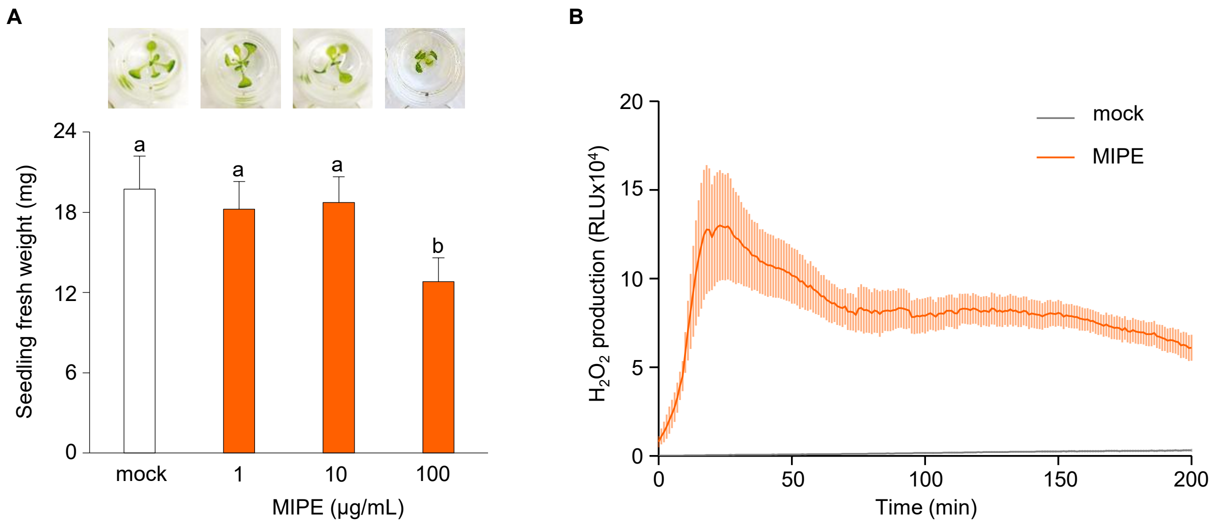

Figure S7. MIPE dose-response on Arabidopsis growth and MIPE induced H_2O_2 production A) Effects of mock or MIPE at different doses on 10-days-old Arabidopsis seedlings growth. (B) Kinetics of H_2O_2 production measured by luminol reaction for 200 min after treatment with sterile distilled water (mock), or MIPE (1 µg/mL) in four-week-old Arabidopsis leaf discs and reported as Relative Luminescence Units (RLU). Data represent mean ± SE (n=6). Data shown represent the mean ±SD (n=10). The experiments were repeated three times with similar results. The different letters indicate significantly different datasets according to ANOVA followed by Tukey's test ($p \leq 0.05$).

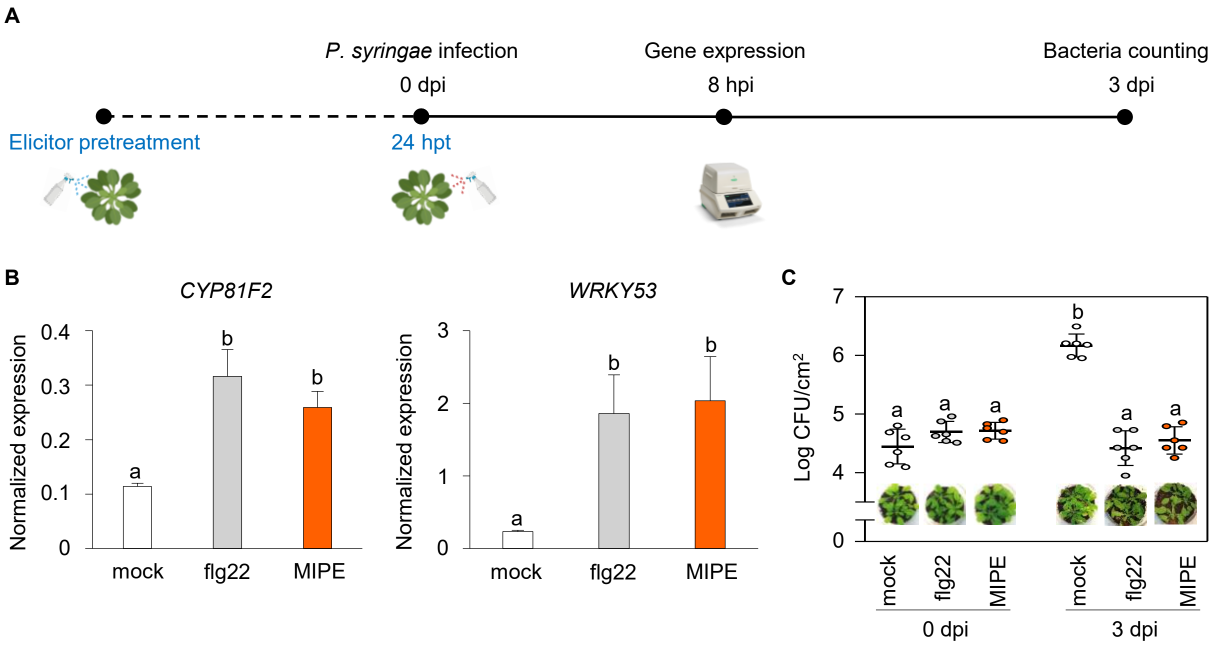

Figure S8. Pre-treatment of Arabidopsis with MIPE enhanced immune response and protection against *P. syringae*. (**A**) Four-week-old Arabidopsis leaves were pretreated with sterile distilled water (mock), flg22 (1 µM), or MIPE (1 µg/mL), and 24 hours post pretreatment (hpt) were inoculated with *P. syringae*.(**B**) Quantitative RT-PCR analysis of *CYP81F2* and *WRKY53* gene expressions in *P. syringae*-infected leaves collected at 8 hours post infection (hpi). mRNA expression levels are normalized to *UBQ5* and *TUB4* expression levels. Data represent the mean ±SE (n=3). (**C**) Colony forming units (Log CFU) of *P. syringae* per leaf area (cm^2) were determined at 0 and 3 days post infection (dpi). Data represent mean ±SD (n=6). The experiments were performed three times with similar results. Different letters indicate significant differences according to ANOVA followed by Tukey's test ($p \leq 0.05$).
